# International university rankings: composite indicators and methodological approaches

**DOI:** 10.3389/frma.2026.1828850

**Published:** 2026-05-25

**Authors:** Konstantinos Latifis, Antonis Sidiropoulos, Georgios Evangelidis

**Affiliations:** 1 Department of Information and Electronic Engineering, International Hellenic University, Sindos, Greece; 2 Department of Applied Informatics, University of Macedonia, Thessaloniki, Greece

**Keywords:** aggregated ranking, composite indicators, Data Envelopment Analysis (DEA), latent-dimension ranking, Pareto dominance, Rainbow Ranking, skyline ranking, university ranking

## Abstract

This article explores the methodological landscape of international university rankings by reconstructing and formalizing the computational pipelines of five widely used global systems (ARWU-ShanghaiRanking, Times Higher Education (THE), U.S. News Best Global Universities, QS World University Rankings, CWUR) and reviews mathematically principled alternatives to fixed-weight composite rank tables. For the major ranking systems, the study details indicator design, normalization and transformation mechanisms and weighted aggregation into final composite scores, highlighting how these design choices encode normative assumptions and drive cross-system discrepancies. Responding to critiques of single-score rank tables, the paper reviews non-aggregative and minimally aggregative paradigms that preserve multidimensionality and reduce misleading ordinal precision, including dominance-based methods (Pareto front, skyline and discriminative skyline ranking), categorical layering (Rainbow Ranking and RR-index), dashboard and group-based multidimensional systems (U-Multirank, CHE-style reporting, and CWTS Leiden Ranking), frontier efficiency models (DEA/FDH), and data-driven latent-dimension approaches (PCA, factor and Bayesian latent-trait models). The article also situates aggregated-ranking voting rules and weighted voting power indices as complementary formal tools for preference aggregation and influence analysis. Overall, it argues that global rankings should be interpreted as algorithmic evaluative frameworks rather than neutral measurements, and that dominance-, profile-, frontier-, and latent-variable approaches offer transparent and conceptually robust complements for multidimensional university evaluation. We aim to identify ranking methods that operate without human-chosen weights or subjective parameter choices, because such choices influence and often distort the resulting rankings.

## 1 Introduction

### 1.1 Background and motivation

Global university rankings have become highly visible instruments in higher education governance, public communication, and institutional strategy. They shape student choices, influence national policy agendas, affect international partnerships and are increasingly incorporated into internal university decision-making as informal performance benchmarks. Their practical appeal lies in a powerful simplification: they compress multidimensional and heterogeneous evidence—research output, citation impact, reputation, internationalization, teaching-related proxies, resources and outcomes—into a comparable score and, often, into a single ordinal position. However, this simplification is also the main methodological problem. Ranking systems do not merely “measure” a pre-existing and universally agreed construct of quality; they operationalize a model of institutional excellence through indicator selection, normalization, scaling, and aggregation choices that embed normative assumptions and statistical compromises (Hazelkorn, 2015; Marginson, 2014; Dill and Soo, 2005; Moed, 2017).

Leading global ranking systems (e.g., ARWU, THE, QS, U.S. News Best Global Universities, and CWUR) vary in their data sources and sets of indicators, yet they generally rely on a shared computational logic: raw indicators are first transformed to make them more comparable across different scales and distributions, and then combined using fixed-weight composite formulas. This framework is methodologically efficient and easy to communicate, but it is also sensitive to seemingly technical design decisions. Choices regarding non-linear compression, standardization, percentile conversion, top-referenced scaling, and weight allocation can significantly affect institutional positions, while the resulting rank tables often encourage over-interpretation of small score differences (Docampo, 2013; Saisana et al., 2011). Consequently, divergence among rankings should not automatically be treated as noise; rather, it frequently reflects distinct evaluative theories embedded in the algorithms themselves.

At the same time, recent research has increasingly moved beyond single-score rank tables. These approaches question whether multidimensional university performance should be expressed as a single ordered ranking. They include non-aggregative dashboards, categorical or tiered systems, dominance-based models grounded in Pareto semantics, frontier-efficiency methods, and latent-dimension techniques, some of which infer data-driven rather than designer-specified weights. Collectively, these frameworks seek to preserve more of the structure of the underlying data, reduce false precision, and make evaluative assumptions more transparent (Brankovic et al., 2018; Johnes, 2015; Stoupas et al., 2021).

### 1.2 Scope and objectives

This article reviews and formalizes the methodological landscape of global university ranking systems with two complementary objectives, through a structured methodological synthesis. First, it reconstructs the computational pipelines of five widely used composite global rankings—ARWU (ShanghaiRanking), Times Higher Education (THE), QS World University Rankings, U.S. News Best Global Universities, and CWUR—by making explicit the sequence of operations from raw indicators to final institutional scores. Emphasis is placed on indicator design, preprocessing, normalization/transformation, and weighted aggregation, because these stages are where normative and statistical assumptions are concretely encoded. In this terms, rankings are treated as algorithmic evaluative frameworks rather than as neutral representations of institutional quality. Different ranking models are therefore understood not merely as alternative measurement techniques, but as alternative evaluative constructions that foreground different dimensions of institutional performance and, in doing so, generate different strategic environments for universities.

Second, the article surveys mathematically principled and methodologically representative alternatives to fixed-weight composite rankings, with particular attention to methods that avoid or minimize human-chosen weights and subjective parameterization. The motivation is not merely technical elegance. In university ranking, exogenously selected weights and compensatory aggregation rules can substantially influence outcomes and distort interpretation when stakeholders derive a meaningful ordering from small numerical differences. The review therefore focuses on approaches that either replace aggregation with partial-order logic or profile-based reporting, or derive structure from data and production frontiers rather than from predetermined weight vectors (Stoupas et al., 2021; Moed, 2017; Emrouznejad and Yang, 2018).

This does not imply that methodological design choices disappear when fixed weights are removed. Selecting which method to apply (e.g., DEA vs. PCA), or the indicator set used to define a Pareto front is itself a normative decision and therefore not neutral. Hence, “weight-free” does not mean value-neutral, but instead it means reducing one major source of arbitrariness—externally imposed fixed weights and compensatory aggregation—while keeping methodological assumptions explicit and inspectable.

In this sense, the reviewed alternatives follow two broad strategies. Dominance-, frontier-, and profile-based approaches avoid imposing a single universal trade-off structure by replacing global aggregation with partial orders, tiers, efficiency frontiers, or multidimensional reporting. Latent-dimension approaches, by contrast, retain a scalar representation but infer the effective aggregation structure from the data through estimated loadings or latent traits. In both cases, the intent is not to eliminate normativity, but to make the evaluative commitments more transparent, auditable, and amenable to robustness and sensitivity analysis.

**Figure 1:**
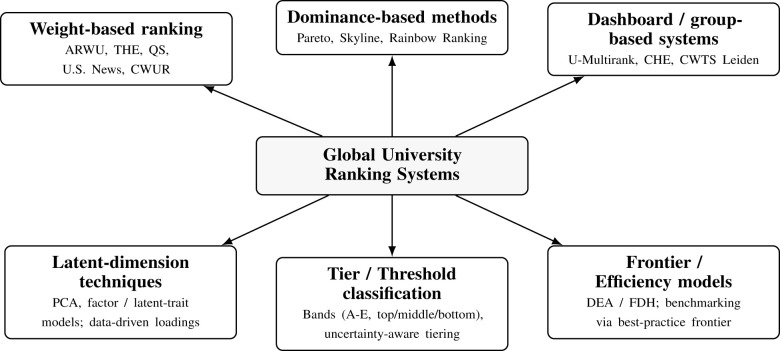
Conceptual map of ranking paradigms: composite systems and neighboring methodological families.

The conceptual map represented in Figure 1 situates conventional weight-based systems alongside surrounding methodological families, thereby highlighting that “global ranking” is not a single technique but a broad design space of competing evaluative logics.

This article is conceived as a narrative, curated methodological review rather than as a PRISMA-style systematic review or a formal scoping review. Its purpose is not exhaustive bibliographic coverage, but a structured analytical synthesis of the principal ranking approaches most relevant to university evaluation. The approaches discussed were selected on the basis of their methodological maturity, conceptual distinctiveness, documented use or clear applicability in higher education, and relevance to the article's central concern with how multidimensional institutional performance is transformed into rank, tier, profile, frontier, or latent score. Accordingly, the review is selective by design and intended as a structured methodological synthesis rather than as an exhaustive literature-mapping exercise.

### 1.3 Methodological perspective and key questions

A central premise of this work is that ranking methodology should be analyzed at the level of formal representation and algorithmic transformation. The relevant question is not only which indicators are used, but how they are rendered commensurable, how trade-offs are authorized, and what type of output structure is ultimately produced (total order, partial order, tiers, profiles, efficiency frontier, or latent score). This perspective clarifies why ranking systems with superficially similar indicators may produce different institutional outcomes, and why methodological transparency is crucial for valid interpretation (Hazelkorn, 2015; Piro and Sivertsen, 2016; Moed, 2017).

Within this perspective, this review addresses four interconnected questions. How do major global rankings achieve comparability across heterogeneous indicators through standardization, scaling, and non-linear transformations? How do fixed weights and composite formulas shape the meaning of the final score and the resulting order? Which alternative frameworks can preserve multidimensionality or reduce compensatory assumptions while remaining computationally feasible and interpretable? And, among these alternatives, which methods can operate without explicit human-assigned weights or strong subjective parameter choices?

These questions are particularly salient in the context of publication- and citation-based evaluation. Bibliometric indicators provide a scalable and externally observable basis for cross-institution comparison, but they also inherit known biases related to field structure, database coverage, language and other publication/citation practices. When such indicators are embedded in composite systems, the effects of preprocessing and aggregation become even more consequential (Piro and Sivertsen, 2016; Moed, 2017). For this reason, this work does not treat the ranking problem as a purely descriptive exercise, but as a modeling problem.

A second, often under-emphasized issue is that ranking outputs—composite, dominance-based, frontier-based, or latent-dimension—inherit the limitations of their input data. In particular, institutional self-reported statistics may suffer from missing values, inconsistent definitions across jurisdictions, limited auditability, and incentives for strategic reporting; bibliometric inputs depend on database coverage, the accurate attribution of publications to institutions and field/language representation. Consequently, methodological sophistication cannot compensate for systematically biased or low-quality primary data: improvements in ranking methodology must be paired with explicit data-quality protocols (verification, harmonized definitions, missing-data handling, and robustness checks) to avoid producing precisely computed but substantively misleading results.

### 1.4 Review structure and main contributions

This review is organized to move from the dominant composite-ranking paradigm to alternative minimally aggregative frameworks, while maintaining a common methodological formalization, comparability, and interpretability. It begins with a detailed examination of five influential global ranking systems and reconstructs their computational pipelines, including indicator definitions, normalization schemes, and weighted score construction. This opening analysis also situates conventional composite rank-table systems in contrast with dashboard-style and group-based multidimensional systems such as U-Multirank, CHE-style reporting, and the CWTS Leiden Ranking, which avoid imposing a universal aggregate order.

The subsequent sections examine major families of alternatives to fixed-weight composite scoring. Dominance-based methods (Pareto fronts, skyline ranking, skyline layering, and related refinements such as discriminative skyline scoring and Rainbow Ranking) are presented as approaches that replace fixed trade-offs with partial comparability and dominance-induced stratification (Borzsonyi et al., 2001; Stoupas et al., 2018, 2021). The discussion then turns to non-aggregative profile and tier frameworks, emphasizing dashboard reporting, categorical performance grouping, and uncertainty-aware classification. A further section surveys frontier and efficiency-based methods (DEA and FDH), which evaluate universities relative to empirically constructed best-practice frontiers rather than through exogenous weighted composites (Johnes, 2015; Emrouznejad and Yang, 2018). It also covers latent-dimension and data-driven reduction approaches (PCA, factor analysis, and Bayesian latent-variable models), which infer implicit aggregation structures from covariance patterns (Brankovic et al., 2018; Visbal-Cadavid et al., 2020). Finally, it discusses voting and aggregated-ranking models from computational social choice as complementary tools for combining rankings and analyzing influence in weighted decision environments (Brandt et al., 2016).

The contribution of this work is primarily methodological and synthetic. It provides a unified, formalized account of how prominent global rankings compute scores and how alternative ranking paradigms can be interpreted within a common comparative framework. The purpose of this review is not to promote one universally superior ranking method, but to encourage more transparent, context-aware, and methodologically plural evaluation practices. In this view, rankings should be interpreted as formal evaluative models whose outputs are meaningful only in relation to their assumptions, rather than as self-evident measurements of a singular and objective institutional quality (Hazelkorn, 2015; Moed, 2017; Marginson, 2014).

### 1.5 Governance effects of university rankings

Global university rankings should be regarded not only as descriptive instruments of comparison but also as governance devices that shape how universities are observed, evaluated, and steered. Once rankings become publicly visible benchmarks for prestige, funding, international positioning, and strategic legitimacy, they begin to influence institutional agendas well beyond their original informational purpose. In this sense, rankings do not merely register differences among universities; they also participate in constructing competitive fields, stabilizing particular definitions of excellence, and directing organizational attention toward those dimensions that are most readily measured and compared. The significance of rankings therefore lies not only in their statistical architecture, but also in their capacity to reframe evaluation as an ongoing regime of public comparison and performance management (Espeland and Sauder, 2007; Brankovic et al., 2023). Different ranking models do not simply provide alternative descriptions of the same institutional world. By privileging different indicators, aggregation rules, and output structures, they help produce different institutional realities, each of which defines excellence, visibility, and strategic relevance in a distinct way.

This view also clarifies why methodological choices have institutional consequences. When indicators become targets, universities may adapt hiring strategies, publication behavior, internationalization policies, internal resource allocation, and reporting practices in order to improve their measurable standing. The problem is not simply that rankings may be statistically imperfect, but that they can generate reactive and sometimes isomorphic responses, encouraging institutions to emulate highly ranked models even when these are misaligned with local missions or disciplinary diversity (Espeland and Sauder, 2007).

From this perspective, methodological critique cannot stop at normalization, weighting, or aggregation formulas, but should also examine the kinds of organizational behavior that ranking systems reward, the forms of diversity they suppress, and the incentives they generate for strategic adaptation. This dynamic can be understood through Goodhart's Law: once an indicator becomes a target of institutional strategy, it may cease to serve as a reliable measure of the underlying academic quality it was originally intended to capture. This is one reason why multidimensional profiles, dominance-based methods, and uncertainty-aware approaches matter. Although they do not eliminate normativity, they can reduce the regulatory force of a single scalar hierarchy, make evaluative trade-offs more explicit, and allow institutional diversity to remain visible. In this sense, the choice among ranking architectures is also a choice among institutional incentive structures, since different evaluative designs encourage different forms of strategic adaptation, organizational visibility, and policy response.

## 2 Global university ranking systems

Global university ranking instruments can be organized into two broad families: major weight-based composite systems and dashboard-style systems that avoid a universal aggregate order. Together, these families capture the dominant ways in which global university performance is translated into comparable evaluative outputs.

The discussion first reconstructs the computational logic of five prominent composite rankings—ARWU (ShanghaiRanking), Times Higher Education (THE), U.S. News Best Global Universities, QS World University Rankings, and the Center for World University Rankings (CWUR)—in order to make explicit how indicator choice, normalization, scaling, and aggregation shape their final results to measuring and comparing institutional performance worldwide. It then examines dashboard-oriented systems, especially U-Multirank, CHE-style and CWTS Leiden Ranking, which retain multidimensional reporting and thus provide an important contrast to fixed-weight composite models.

### 2.1 Weight-based university ranking systems

#### 2.1.1 ARWU (ShanghaiRanking)

Academic Ranking of World Universities (ARWU) is a typical example of a research-intensive, externally sourced ranking system. It operationalizes institutional performance primarily through high-end research achievements and bibliometric output, largely avoiding institutional self-reporting and reputation surveys. Institutional research performance is quantified using the ARWU indicator set and the corresponding weighting scheme reported in Table 1. ARWU's computational pipeline is well-documented in secondary reconstructions that validate its reproducibility and its characteristic non-linearity (Docampo, 2013; Massucci and Docampo, 2019).

**Table 1:** ARWU indicators, codes, and weighting scheme.

Criteria	Indicator	Code	Weight
Quality of education	Alumni of an institution winning Nobel Prizes and Fields Medals	Alumni	10%
Quality of faculty	Staff of an institution winning Nobel Prizes and Fields Medals	Award	20%
	Highly cited researchers	HiCi	20%
Research output	Papers published in *Nature* and *Science*	N&S	20%
	Papers indexed in SCI-Expanded and SSCI (Web of Science)	PUB	20%
Per capita performance	Per capita academic performance of an institution	PCP	10%

Let *R*_*i, j*_ denote the raw value for university *i* on indicator *j* (e.g., Alumni, Award, HiCi, N&S, PUB, PCP). ARWU applies a non-linear compression to reduce the skewness typical of award and citation distributions via square-root scaling. The ARWU transformation, per-capita adjustment, and final aggregation are formalized in Equations 1–4:


$$\begin{array}{l} S_{i,j}=100\cdot \frac{\sqrt{R_{i,j}}}{\sqrt{R_{\max,j}}} \end{array}$$ (1)

where *R*_max, *j*_ is the global maximum for indicator *j* (Docampo, 2013). The per-capita performance (PCP) indicator is constructed by combining the squared transformed indicator scores into a weighted sum and normalizing by full-time equivalent staff (FTE), followed by a rescaling to a 0–100 score:


$$\begin{array}{l} WSS_{i}=0.1S_{i,Alumni}^{2}+0.2\left(S_{i,Award}^{2}+S_{i,HiCi}^{2}+S_{i,N\&S}^{2}+S_{i,PUB}^{2}\right) \end{array}$$ (2)

$$\begin{array}{l} {\tilde{PCP}}_{i}=\sqrt{\frac{WSS_{i}}{FTE_{i}}},\;S_{i,PCP}=100\cdot \frac{{\tilde{PCP}}_{i}}{\max_{k}{\tilde{PCP}}_{k}} \end{array}$$ (3)

Finally, ARWU aggregates the six standardized indicator scores through a fixed-weight linear combination:


$$\begin{array}{l} Total_{i}=0.1S_{i,Alumni}+0.2S_{i,Award}+0.2S_{i,HiCi}+0.2S_{i,N\&S}+\\ \;0.2S_{i,PUB}+0.1S_{i,PCP} \end{array}$$ (4)

The result is then normalized so that the top institution receives 100 and others are expressed relative to this benchmark. Methodologically, ARWU's strengths are its reliance on externally verifiable inputs and its relative stability over time (partly due to the square-root compression), while its principal limitation is conceptual: it privileges a narrow, research-elite notion of excellence and systematically under-represents teaching, local mission, and many disciplinary profiles, particularly outside citation-intensive STEM fields (Piro and Sivertsen, 2016; Hazelkorn, 2015). ARWU therefore tends to favor institutions with historically accumulated prestige and elite research distinction, framing university excellence primarily in terms of globally visible research achievement.

#### 2.1.2 Times Higher Education (THE) World University Rankings

Times Higher Education (THE) World University Rankings represent a multidimensional framework built on five pillars: *Teaching: 29.5%* (teaching reputacion, student staff ratio, number of doctoral degrees, doctoral bachelor ratio, institutional income), *Research Environment: 29%* (university's reputation for research excellence, research income, research productivity), *Research Quality: 30%* (citation impact, research strength, research excellence, research influence), *International Outlook: 7.5%* (proportion of international students, proportion of international academic staff, level of international collaboration in research publications, study abroad), *Industry Income: 4%* (collaboration between academy and industry, partnerships and technology transfer agreements, number of patents of the university) (Times Higher Education, 2025).

In contrast to ARWU, THE explicitly combines bibliometric evidence (primarily via Scopus) with reputation surveys and a set of institutionally supplied and audited statistics (e.g., staffing, income, doctoral output). This architecture aims to approximate multiple missions of universities, yet it also introduces challenges of transparency and measurement comparability, especially where proprietary cleaning and survey-weighting procedures are not fully disclosed (Harvey, 2008; Hazelkorn, 2015).

A core statistical feature of THE is the standardization of heterogeneous indicators into *z-scores*. The THE standardization and aggregation pipeline is summarized in Equations 5–8. For an indicator value *x*, THE computes:


$$\begin{array}{l} z=\frac{x-\mu }{\sigma } \end{array}$$ (5)

where μ and σ are the global mean and standard deviation for that indicator (Saisana et al., 2011). The standardized values are then mapped to a 0–100 scale via a min–max transformation on the z-score range:


$$\begin{array}{l} S=100\times \frac{z-z_{\min}}{z_{\max}-z_{\min}} \end{array}$$ (6)

where *z*_max_ = the top-performing university's z-score, *z*_min_ = the lowest-performing university's z-score. The weighted score for each indicator is computed as:


$$\begin{array}{l} Score_{i}=w_{i}\times S_{i} \end{array}$$ (7)

where *w*_*i*_ = the indicator weight, *S*_*i*_ = the standardized score for indicator *i* according to its importance within THE's five-pillar framework. THE publishes the detailed weighting distribution annually. The Final Institutional Score is calculated as the weighted sum of all 18 sub-indicator scores:


$$\begin{array}{l} FinalScore=\overset{18}{\underset{i=1}{\sum }}w_{i}S_{i} \end{array}$$ (8)

The top-ranked university in the dataset is assigned a final score of 100, and all other institutions receive a proportionally scaled score. This ensures comparability across ranking cycles and provides a stable reference point for longitudinal evaluations.

In conceptual terms, THE's multidimensionality is a key advantage as it does not reduce “quality” of research output. The use of Field-weighted citation impact (FWCI) as a bibliometric indicator normalizes citations across disciplines, allowing smaller institutions or those with strong humanities and social science outputs to compete effectively with large STEM-focused universities. Nevertheless, institution size normalization reduces size related bias (Times Higher Education, 2025), while regional weighting in survey results mitigates overrepresentation of spesific regions/countries. However, the heavy influence of reputation survey components, together with the dependence on database coverage and English-language publication visibility, can reinforce existing prestige hierarchies and disadvantage institutions with strong local or teaching missions (Marginson, 2014; Hazelkorn, 2015). THE therefore broadens the evaluative frame beyond research output, but does so through a model that is increasingly mediated by composite proxies and reputational inputs, yielding a broader yet also more normatively structured and partially opaque construction of institutional excellence.

#### 2.1.3 Quacquarelli Symonds (QS) World University Rankings

Quacquarelli Symonds World University Rankings is among the most visible global rankings and is characterized by a strong reputational component combined with quantitative proxies for research influence, teaching capacity, and internationalization (Sowter et al., 2017). Its methodology integrates large-scale academic and employer reputation surveys with bibliometric data (Scopus) and institutionally reported counts (e.g., faculty and students), complemented in recent iterations by additional metrics such as sustainability, employment outcomes, and international research networks (QS Quacquarelli Symonds, 2025). The methodology adopted by QS proceeds through (i) data acquisition and validation, (ii) indicator computation and normalization, (iii) statistical scaling and transformation of scores and (iv) weighted aggregation into the final ranking index.

Here the indicators concern: academic reputation—30%, employer reputation—15%, citations per faculty—20%,faculty–student ratio—10%, international faculty ratio—5% and international student ratio—5%, employment outcomes—5%, international research network—5% and sustainability—5%. The QS reputation scoring, indicator normalization, and final composite aggregation are formalized in Equations 9–13. First the reputation score for institution *i* is defined as:


$$\begin{array}{l} R_{i}=\overset{N}{\underset{j=1}{\sum }}w_{j}\cdot s_{ij} \end{array}$$ (9)

where *s*_*ij*_ represents the nominations made by respondent *j* and *w*_*j*_ denotes the jointly applied geographic and disciplinary weighting factor. QS scales reputation results through min–max normalization:


$$\begin{array}{l} R_{i}^{\prime }=100\times \frac{R_{i}-\min\left(R\right)}{\max\left(R\right)-\min\left(R\right)} \end{array}$$ (10)

Separate computations are carried out for academic reputation *R*_acad, *i*_ (30%) and employer reputation *R*_empl, *i*_ (15%).

For citations per faculty, QS combines field-normalized citation counts with a faculty denominator:


$$\begin{array}{l} C_{\text{norm},i}=\overset{F}{\underset{k=1}{\sum }}\frac{C_{ik}}{E\left[C_{k}\right]},\;CPF_{i}=\frac{C_{\text{norm},i}}{FTE_{i}},\\ \;CPF_{i}^{\prime }=100\times \frac{CPF_{i}-\min\left(CPF\right)}{\max\left(CPF\right)-\min\left(CPF\right)} \end{array}$$ (11)

$$\begin{array}{l} \;FSR_{i}=\frac{{\text{Faculty}}_{i}}{{\text{Students}}_{i}},\\ FSR_{i}^{\prime }=100\times \frac{\log\left(FSR_{i}\right)-\min\left(\log\left(FSR\right)\right)}{\max\left(\log\left(FSR\right)\right)-\min\left(\log\left(FSR\right)\right)} \end{array}$$ (12)

Accordingly for institution *i* QS define the other indicators


$$IFR_{i},ISR_{i},EO_{i},IRN_{i},SUST_{i}$$

The final QS composite is a fixed-weight sum over normalized indicators (with weights adjusted following recent methodological expansions):


$$\begin{array}{l} Q_{i}=0.30R_{\text{acad},i}+0.15R_{\text{empl},i}+0.20CPF_{i}^{\prime }+0.10FSR_{i}^{\prime }\\ \;+0.05IFR_{i}^{\prime }+0.05ISR_{i}^{\prime }+0.05EO_{i}^{\prime }+0.05IRN_{i}^{\prime }+0.05SUST_{i}^{\prime } \end{array}$$ (13)

QS's principal strength is its breadth and its communicative salience for multiple stakeholders, particularly prospective students and employers. Its principal limitation is methodological: a high share of the score derives from reputation surveys and partially opaque adjustments (e.g., respondent weighting, outlier dampening), which reduces reproducibility and can entrench historical hierarchies (Dill and Soo, 2005; Hazelkorn, 2015). Despite these limitations, QS remains one of the most globally visible and widely used systems of institutional comparison. At the same time, its strong reliance on reputational and proxy-based indicators means that the system tends to reward institutions that are already highly visible within global academic and employer perception networks, thereby reinforcing a more perception-mediated construction of institutional quality.

Recent methodological developments also show that sustainability has moved from a peripheral concern to a more explicit evaluative dimension in global rankings. This is especially visible in the emergence of the QS Sustainability Rankings, which extend the QS ranking ecosystem beyond the traditional emphasis on reputation, research influence, and internationalization. In this framework, sustainability is not treated merely as a minor auxiliary indicator but as a dedicated domain of institutional assessment structured around environmental impact, social impact, and governance. As a result, universities are increasingly evaluated not only as academic competitors, but also as organizations expected to demonstrate measurable commitments to climate action, inclusion, social responsibility, and institutional accountability (Buckner and Zhang, 2025).

A parallel but somewhat different development is represented by UI GreenMetric, initiated by Universitas Indonesia in 2010 as one of the earliest global sustainability-focused university rankings (Alberti et al., 2025). Whereas QS Sustainability reflects the expansion of an established global ranking brand toward ESG- and SDG-related assessment, UI GreenMetric has historically been more campus-centered, emphasizing sustainability in infrastructure, energy and climate change, waste, water, transportation, and education and research, with recent extensions toward governance-oriented dimensions. The rise of such frameworks signals a broader methodological shift in university evaluation: indicator selection increasingly encodes explicit normative assumptions about what universities should contribute to society, not only how they perform in research-intensive competition. At the same time, recent literature suggests that sustainability rankings may reproduce familiar ranking problems, including administrative burden, standardization pressures, limited contextual sensitivity, and the risk that signaling and visibility may be rewarded alongside substantive institutional transformation (Buckner and Zhang, 2025; Alberti et al., 2025).

#### 2.1.4 U.S. News and World Report (best global universities rankings)

Published annually by *U.S. News and World Report* since 2014, the *best global universities rankings* are primarily oriented toward comparative research performance and is grounded in bibliometric data sourced from Clarivate Analytics, using the *Web of Science* and *InCites* platforms (US News and World Report, 2025). Its design differs from THE and QS in its avoidance of institutional self-reported data. Instead, it relies on externally sourced metrics and employs a distributional scoring logic that is less sensitive to extreme skewness than raw count measures. While reputation indicators appear in the form of Clarivate-administered survey components, the overall framework remains predominantly bibliometric in structure (Hazelkorn, 2015; Rauhvargers, 2013).

The methodology uses ten fixed-weight indicators: publications (10%), books (2.5%), citations (7.5%), global research reputation (12.5%), regional research reputation (12.5%), conferences (2.5%), top 10% most cited publications (22.5%), top 1% most cited papers (10%), international collaboration (10%), and normalized citation impact (10%). The percentile transformation, weighted composite score, and final normalization used in the U.S. News model are given in Equations 14–16. After indicator-specific values are obtained, raw values are converted into percentile scores reflecting each institution's standing relative to all other institutions with valid data:


Pi(k)=rank of university i on indicator k Nk (14)

where $P_{i}^{\left(k\right)}$ denotes the percentile score of university *i* on indicator *k*, and *N*_*k*_ is the number of institutions with valid data for that indicator. This percentile-based transformation reduces the influence of extreme skewness in bibliometric distributions.

The final composite score is then computed as a weighted sum of the percentile scores:


$$\begin{array}{l} FinalScore_{i}=\overset{10}{\underset{k=1}{\sum }}w_{k}P_{i}^{\left(k\right)} \end{array}$$ (15)

where *w*_*k*_ denotes the fixed weight assigned to indicator *k*. The resulting score is normalized so that the highest-scoring university receives 100:


$$\begin{array}{l} Score_{i}=100\times \frac{FinalScore_{i}}{FinalScore_{\max}} \end{array}$$ (16)

A distinctive property of this model is its strong emphasis on citation excellence through top-percentile publication indicators, especially the top 10% and top 1% most cited papers. Methodologically, this improves sensitivity to high-impact research and supports cross-field comparability when indicators are properly normalized by field and year (US News and World Report, 2025). However, the system's principal limitation is scope. The methodology has been critiqued for underrepresenting important aspects of higher education such as teaching quality, student learning, and societal engagement, while focusing exclusively on bibliometric indicators (Hazelkorn, 2015). U.S. News therefore functions more convincingly as a benchmark of globally visible research performance than as a comprehensive measure of university quality.

#### 2.1.5 Center for World University Rankings (CWUR)

*Center for World University Rankings* (CWUR), founded in 2012 and operating from the United Arab Emirates and the United States, publishes one of the few global university rankings based exclusively on objective and measurable indicators. Unlike QS or THE, CWUR does not employ reputation surveys or institutional self-reporting. Instead, it relies entirely on independently verifiable datasets related to alumni achievements, faculty distinctions, and research performance. CWUR positions itself as an objective, outcome-based alternative to survey-heavy models. It avoids reputation surveys and institutional self-reporting, relying instead on third-party datasets for alumni achievements and employment outcomes, faculty distinctions, and research performance (Center for World University Rankings, 2025).

Its indicator framework uses seven indicators, assigning substantial weight to alumni education and employment outcomes, thereby operationalizing institutional impact through observable downstream achievements rather than process measures. The CWUR normalization and weighted aggregation scheme is formalized in Equations 17–20. Table 2 provides an overview of the seven CWUR indicators, their corresponding weights, and their schematic mathematical representation.

**Table 2:** WUR indicators and their weighting scheme.

Indicator	Description	Weight
Quality of education	Weighted count of alumni earned major international awards, normalized by institutional size.	25%
Alumni employment	Number of alumni holding executive leadership roles in major global corporations or government.	25%
Quality of faculty	Faculty members receiving significant international distinctions and prizes.	10%
Research output	Total volume of publications indexed in Web of Science or Scopus.	10%
High-quality publications	Publications appearing in top-tier journals such as *Nature* and *Science*.	10%
Influence	Publications in high-impact journals, calculated using journal impact factors.	10%
Citations	Total citations received across the institution's indexed publications.	10%

For university *i*, let *QE*_*i*_, *AE*_*i*_, *QF*_*i*_, *RO*_*i*_, *HQP*_*i*_, *INF*_*i*_, and *CIT*_*i*_ denote, respectively, the raw scores for *Quality of Education, Alumni Employment, Quality of Faculty, Research Output, High-Quality Publications, Influence*, and *Citations*. CWUR typically applies maximum-based normalization, that is, normalization relative to the top-performing institution. For an indicator value *X*_*i*_ and the corresponding maximum *X*_max_, the normalized score is written as


$$\begin{array}{l} X_{i}^{\prime }=100\times \frac{X_{i}}{X_{\max}}. \end{array}$$ (17)

More specifically, *Quality of Education* and *Alumni Employment* (normalized by size) may be represented as


$$\begin{array}{l} QE_{i}=\frac{A_{i}}{E_{i}},\;AE_{i}=\overset{N_{i}}{\underset{j=1}{\sum }}w_{j} \end{array}$$ (18)

where *A*_*i*_ denotes the weighted count of distinguished alumni achievements, *E*_*i*_ is a size-related normalization term, *w*_*j*_ denotes the weight associated with the prominence of the executive or leadership position held by alumnus *j* and *N*_*i*_ is the number of such alumni counted for institution *i*.

The remaining indicators are represented schematically as follows:


$$\begin{array}{l} QF_{i}=F_{i},\;RO_{i}=P_{i},\;HQP_{i}=H_{i},\;INF_{i}=I_{i},\\ \;CIT_{i}=C_{i}, \end{array}$$ (19)

where *F*_*i*_ denotes distinguished faculty achievements, *P*_*i*_ the total indexed publication output, *H*_*i*_ the volume of publications in highly selective journals, *I*_*i*_ the volume of publications in influential journals, and *C*_*i*_ the total citations received.

Let $QE_{i}^{\prime }$, $AE_{i}^{\prime }$, $QF_{i}^{\prime }$, $RO_{i}^{\prime }$, $HQP_{i}^{\prime }$, $INF_{i}^{\prime }$, and $CIT_{i}^{\prime }$ denote the normalized scores of university *i* in these seven dimensions. The final CWUR score is then given by the weighted sum of the normalized indicators:


$$\begin{array}{l} CWUR_{i}=0.25QE_{i}^{\prime }+0.25AE_{i}^{\prime }+0.10QF_{i}^{\prime }+0.10RO_{i}^{\prime }\\ \;+0.10HQP_{i}^{\prime }+0.10INF_{i}^{\prime }+0.10CIT_{i}^{\prime } \end{array}$$ (20)

CWUR's principal advantage is its independence from institutional submissions and its reduced exposure to survey bias. At the same time, its strong emphasis on alumni employment prominence and award-based achievements may reflect labor-market structures, historical reputation, and national economic positioning as much as institutional performance. Scholars have therefore argued that CWUR may privilege older, research-intensive, or well-funded universities, while underrepresenting institutions that are strong in fields such as the humanities or social sciences (Piro and Sivertsen, 2016). Even so, CWUR remains useful in contexts requiring strictly measurable outcomes, although it privileges downstream outcomes and external visibility and may underrepresent more context-specific academic missions or less publicly legible forms of institutional contribution.

### 2.2 Dashboard and group-based multidimensional systems

The term *dashboard* refers to systems that present parallel indicator results and support interactive “like-with-like” comparisons without enforcing a universal aggregate score, whereas *group-based* reporting denotes methods that map indicator performance into categories (e.g., A–E bands or top/middle/bottom groups) to avoid misleading ordinal precision. As will be discussed in a later section, both approaches share a non-aggregative design principle: they prioritize indicator-level transparency and stakeholder-driven interpretation over compensatory trade-offs embedded in fixed weighting schemes. Accordingly, the systems discussed below publish results per indicator and dimension—often complemented by filtering, benchmarking, and field- or subject-level views, rather than computing a single composite ordering.

#### 2.2.1 U-Multirank

U-Multirank was initiated under a European policy initiative to provide a transparent, user-driven alternative to global rank tables for higher education, with an emphasis on multidimensional measurement rather than a single composite rank (European Commission, 2014; European Union, 2015). The European Commission supported the development, culminating in the public launch of U-Multirank in 2014 (European Commission, 2014). Early implementation reports describe the first release (May 2014) as covering hundreds of institutions worldwide and combining institutional-level and field-level perspectives (European Union, 2015). Its design explicitly supports stakeholder-driven exploration across institutional and field levels, publishing results per indicator and dimension rather than computing an overall composite ordering.

##### 2.2.1.1 Algorithmic structure: from raw data to performance profiles

U-Multirank implements the dashboard paradigm through a pipeline summarized as:

1. Data acquisition and verification: Indicators are compiled from multiple sources, including institutional data (with verification protocols) and bibliometric or other external datasets where applicable (Jongbloed, 2014).
2. Direction harmonization and normalization: Raw values *x*_*ij*_ are transformed into comparable normalized values *z*_*ij*_ = ϕ_*j*_(*g*_*j*_(*x*_*ij*_)).
3. Performance grouping: Instead of computing a composite score, institutions are assigned to five performance groups (A–E) on each indicator, where A denotes “very good” and E denotes “weak” performance.
4. User-driven comparison: Users explore results through indicator selection, filtering, and like-with-like comparisons rather than accepting an imposed global ordering.

A defining design choice is the use of *indicator-level performance groups* rather than aggregated ranks. Implementation documentation states that U-Multirank “does not provide oversimplified rank tables” and instead assigns A–E groupings per indicator (European Union, 2015).

A characteristic feature of dashboard systems is extensibility: dimensions and indicators can evolve as stakeholder needs and policy goals change. Reports on U-Multirank-related indicator development emphasize expansion toward new dimensions (e.g., teaching and learning effectiveness) while maintaining the non-aggregative design principle (U-Multirank, 2021). This reflects a broader methodological orientation in dashboard rankings: rather than stabilizing a single composite formula, the framework prioritizes transparent reporting and adaptive indicator portfolios.

#### 2.2.2 CHE University Ranking (group-based multidimensional reporting)

The CHE University ranking provides a program- and subject-oriented multidimensional evaluation and explicitly relies on grouping logic to avoid artificial precision. Public documentation describes the use of performance groups (e.g., top/middle/bottom) based on deviation from mean values and confidence intervals, emphasizing that the method avoids presenting illusory fine-grained differences (DAAD, 2025). Equation 21 expresses this group-based classification logic in compact mathematical form. Conceptually, this can be modeled as assigning each institution or program to


$$\begin{array}{l} {\text{Group}}_{ij}\in \left\{\text{Top},\text{Middle},\text{Bottom}\right\} \end{array}$$ (21)

based on statistically meaningful separation rather than composite scoring.

#### 2.2.3 CWTS Leiden ranking

Although research-focused, the CWTS Leiden ranking aligns with the dashboard philosophy by providing multiple bibliometric indicators and emphasizing methodological documentation rather than a single composite index (CWTS Leiden Ranking, 2025a). It supports indicator-level interpretation across multiple dimensions (e.g., impact, collaboration) and has been positioned as a transparent alternative to composite global rank tables.

The CWTS Leiden Ranking offers two editions: the Traditional Edition, which provides bibliometric indicators based on the Web of Science database and draws on more than two decades of methodological development, and the Open Edition, launched in 2024, which provides bibliometric indicators based on the OpenAlex database and emphasizes fully transparent analytics based on open data.

### 2.3 Comparative synthesis and methodological implications

Across ARWU, THE, U.S. News, QS, and CWUR, a shared algorithmic template is visible: heterogeneous indicators are (i) collected from bibliometric infrastructures, surveys, and/or institutional submissions; (ii) standardized through top-referenced scaling, min–max mapping, z-score normalization, or percentile conversion; and (iii) aggregated through fixed weights into a single composite score. The systems differ primarily in their data regimes and in the statistical transformations that mediate skewness and scale comparability. ARWU emphasizes research-elite achievements and stabilizing non-linear compression (Docampo, 2013); THE and QS embed reputational perceptions and broader mission proxies, with the attendant benefits and risks (Hazelkorn, 2015; Marginson, 2014); U.S. News implements a bibliometric benchmark with percentile-based dampening and strong impact sensitivity (US News and World Report, 2025); CWUR foregrounds outcome indicators and third-party data independence (Center for World University Rankings, 2025).

In contrast, dashboard-style systems (e.g., U-Multirank, CHE-style group-based reporting, and the CWTS Leiden ranking) implement a different methodological commitment: rather than enforcing compensatory trade-offs through a fixed aggregation rule, they preserve indicator-level information and communicate institutional performance as a multidimensional profile, often through performance groups or parallel indicator dashboards (European Union, 2015; DAAD, 2025; CWTS Leiden Ranking, 2025a). Algorithmically, their common template replaces the single-score aggregation step described above, with indicator-wise normalization and classification (e.g., A–E bands or top/middle/bottom groups) plus user-driven selection, filtering, and “like-with-like” comparison. This shift changes the interpretive status of the output: from an ordinal rank table that implies total comparability, to a structured set of partial comparisons that foregrounds dimension-specific strengths and weaknesses.

These rankings should not be interpreted as neutral measurements of a single underlying construct, but as structured evaluative models whose outputs depend on normative choices (indicator selection and weights), database coverage (field and language effects), and statistical preprocessing (transformations, outlier handling, and normalization) (Hazelkorn, 2015; Moed, 2017; Piro and Sivertsen, 2016). Consequently, cross-ranking disagreement is often a signal that different systems encode different theories of institutional excellence.

These methodological differences are not merely technical variations; they embody persistent tensions between competing evaluative aims. Composite rankings maximize broad comparability, communicative simplicity, and strategic visibility, but often do so at the cost of construct validity, since heterogeneous institutional missions and outputs are compressed into a single scalar order. By contrast, dashboard- and profile-based systems preserve multidimensionality and reduce false precision, yet they sacrifice the rhetorical force and immediate legibility of a unified rank table. Similar trade-offs arise across the alternative families reviewed below: frontier models gain sensitivity to input–output efficiency but depend strongly on model specification; dominance-based approaches reduce compensatory assumptions but may yield only partial orders; and latent-variable methods replace fixed weights with data-driven structure, while introducing sample dependence and interpretive opacity. In this sense, no ranking architecture simultaneously maximizes comparability, validity, transparency, and multidimensional fidelity. The choice of model therefore always involves an explicit trade-off among partially competing evaluative goods (Dill and Soo, 2005; Hazelkorn, 2015).

These differences are not merely technical. Each ranking architecture embodies a distinct theory of what should count as meaningful institutional performance, how heterogeneous forms of academic activity should be made comparable, and what kinds of evidence are considered sufficiently valid for evaluative use. In this respect, ranking models also favor different modes of accountability, strategic adaptation, and institutional development. Some privilege external visibility and reputational competition, while others support internal diagnosis, peer benchmarking, or efficiency-oriented adjustment. Methodological choices therefore shape not only numerical outcomes, but also the kinds of organizational realities that universities are encouraged to recognize, prioritize, and reproduce (Brankovic et al., 2018; Espeland and Sauder, 2007). Disagreements across models should thus be interpreted not only as noise in measurement, but as an indication that distinct evaluative logics are operating.

Critiques of composite rank tables have motivated alternative ranking paradigms that preserve multidimensionality or reduce compensatory assumptions. Dominance-based and skyline approaches replace fixed trade-offs with partial orders induced by Pareto dominance, yielding tiers (fronts/layers) rather than strict total orders (Stoupas et al., 2021). Profile-and-tier systems such as U-Multirank and CHE-style grouping communicate performance via indicator-level categories rather than a global scalar, thereby reducing false precision and supporting “like-with-like” comparison (European Union, 2015; DAAD, 2025). Frontier (efficiency) methods evaluate institutions relative to a best-practice production frontier (DEA/FDH), deriving efficiency scores from input-output relations rather than from exogenously weighted composites (Johnes, 2015; Emrouznejad and Yang, 2018). Finally, latent-dimension approaches (PCA, factor models, Bayesian latent trait models) estimate implicit weights from covariance structure to derive reduced-dimension indices, at the cost of sample dependence and interpretability challenges (Moed, 2017; Brankovic et al., 2018).

Overall, the dominant global ranking systems can be understood as variations on a composite-indicator pipeline, but their differences should not be reduced to implementation details alone. What separates them is also the balance they strike between comparability, interpretability, validity, and multidimensional representation. Complementarily, dashboard-style systems operationalize a different communication and decision-support logic: they trade the rhetorical simplicity of a single rank table for richer multidimensional reporting, enabling stakeholders to prioritize dimensions differently and to avoid interpreting small scalar differences as substantively meaningful. The growing diversity of alternative ranking systems highlights a central methodological point: ranking is not merely an arithmetic procedure but an explicit modeling decision about which trade-offs are acceptable when multidimensional evidence is represented, compared and communicated. For institutional users, these differences imply that strategic visibility, benchmarking, diagnostic profiling, and uncertainty-aware interpretation require different ranking architectures, which should therefore be treated as purpose-specific evaluative tools rather than as interchangeable measures of overall university quality.

Table 3 summarizes the principal alternative methodological families reviewed below and highlights representative applications to higher-education institutions. At the same time, these families represent more than technical alternatives to composite rankings, since they differ in what they treat as a meaningful basis of comparison and in the kinds of institutional conclusions they license.

**Table 3:** Alternative ranking methods and higher education applications.

Methodological approach	Representative higher-education application	Main evaluative output	Key references
Pareto/skyline ranking	Universities evaluated through non-dominated sorting, with institutions assigned to skyline or Pareto layers rather than forced into a complete rank order.	Pareto fronts/skyline layers	Stoupas et al., 2020, 2021
Rainbow/multidimensional dominance-based ranking	Multidimensional bibliometric comparison of higher-education units without fixed compensatory weights, illustrated for Greek departments of computer science and engineering.	Categorical multidimensional profile / RR-index	Sidiropoulos et al., 2017; Stoupas and Sidiropoulos, 2021
Dashboard/profile systems	User-driven multidimensional profiling of universities through indicator-level comparison rather than a single global league table, as in U-Multirank and the Leiden Ranking.	Indicator dashboard/vector-valued institutional profile	European Union, 2015; Jongbloed, 2014; CWTS Leiden Ranking, 2025a
Tier/threshold/uncertainty-aware classification	Grouping institutions into performance bands or robust categories, including threshold-based classification and uncertainty-aware research evaluation.	Bands, groups, or robust tiers rather than precise ordinal ranks	DAAD, 2025; Abramo et al., 2016; CWTS Leiden Ranking, 2025b
Frontier/efficiency methods (DEA and related non-parametric models)	Estimation of relative efficiency, costs, and heterogeneity in higher education systems, including applications to U.S. and European universities.	Efficiency score relative to a best-practice frontier	Agasisti and Johnes, 2015; Wolszczak-Derlacz, 2017
PCA-based latent reduction	Empirical reinterpretation of university rankings through latent dimensions, including ARWU structure, longitudinal inter-ranking comparison, and PCA-based competitiveness indices.	Principal-component score / reduced-dimension ranking index	Dehon et al., 2010; Selten et al., 2020; Vashchenko et al., 2022
Factor/latent-variable models	University performance assessed through latent constructs, block-balanced factor models, and Bayesian latent-variable estimation of institutional quality.	Factor score/latent-quality estimate/posterior ranking with uncertainty	Visbal-Cadavid et al., 2020; Claassen, 2015; Guarino et al., 2005

## 3 Dominance-based methods (Pareto/skyline family)

### 3.1 Pareto front and non-dominated sorting

Pareto-based evaluation methods constitute a central class of multiobjective optimization techniques that assess universities without imposing externally defined weights or calibration rules. Dominance-based evaluation is mathematically grounded in Pareto optimality. Consider a dataset *D*⊆ℝ^*d*^ describing entities (e.g., universities) by *d* quantitative criteria.

Formally, let a university be represented by a d-dimensional performance vector. Equations 22–24 define the performance-vector representation, Pareto dominance relation, and resulting hierarchy of Pareto fronts.


$$\begin{array}{l} {\text{x}}_{i}=\left(x_{i1},x_{i2},\ldots ,x_{id}\right)\in {\mathbb{R}}^{d} \end{array}$$ (22)

where *x*_*ik*_ denotes the score of university *i* on criterion *k* (e.g., research output, impact, student-to-staff ratio). In this framework, a university **x**_*i*_ is said to *dominate* another university **x**_*j*_ if and only if:


$$\begin{array}{l} x_{ik}\geq x_{jk}\;\forall k\in \left\{1,\ldots ,d\right\}\;\text{and }\exists k:x_{ik}>x_{jk} \end{array}$$ (23)

The set of institutions that are not dominated by any other constitute the *first Pareto front*. Subsequent fronts are generated iteratively by removing previously identified non-dominated sets, yielding a hierarchy:


$$\begin{array}{l} F_{1},F_{2},\ldots ,F_{m} \end{array}$$ (24)

Dominance induces a *partial order* on *D*, allowing incomparability when entities exhibit different trade-off profiles across criteria. This order-theoretic approach provides a principled alternative to weighted composites. Stoupas and Sidiropoulos (Stoupas and Sidiropoulos, 2021) emphasize that such partial orders better reflect the multidimensional character of university performance, arguing that strict total rankings often lack meaning in this context. Pareto frontiers explicitly preserve the plurality of institutional missions as teaching, research, impact, internationalization and societal engagement, without collapsing them into a single scalar indicator. Rather than producing a strict total order, Pareto frontiers yield equivalence classes that reflect trade-offs across competing performance dimensions (Stoupas et al., 2021).

From computational perspective, recent multiobjective optimization research has improved the tractability of Pareto frontier estimation, even in high-dimensional or complex evaluative contexts. Quttineh et al. (2022) introduced efficient approximation techniques for challenging bi-objective systems, demonstrating that modern approaches can broaden the applicability of Pareto methods to large-scale institutional datasets, including international university systems.

From evaluative perspective, Pareto-based rankings exhibit several advantages: (i) they avoid arbitrary weighting and normalization, (ii) they preserve the structure of multidimensional data, (iii) they enhance interpretability by identifying clusters of institutions with comparable trade-off profiles and (iv) they provide a tiered classification scheme suitable for decision-making contexts where relative rather than absolute distinctions are more meaningful. Pareto-based university ranking thus represents a mathematically rigorous and conceptually transparent alternative to traditional weighted systems.

### 3.2 Skyline ranking methods

Skyline-based ranking methods are fundamentally grounded in Pareto optimality and the concept of non-dominance. Given a set of entities evaluated across multiple criteria, an entity belongs to the Skyline if no other entity is at least as good in all dimensions and strictly better in at least one. This principle, originating in multi-objective optimization and order theory, captures the idea that performance is inherently multi-dimensional and that excellence cannot always be expressed through a single linear preference. In this sense, Skyline methods are mathematically equivalent to identifying the first Pareto front, offering a weight-free alternative to conventional ranking models that impose predefined trade-offs among criteria (Borzsonyi et al., 2001; Sidiropoulos et al., 2016; Stoupas et al., 2021).

In contrast to classic weighted composite rankings Skyline-based approaches adopt an order-theoretic perspective. Rather than aggregating indicators, they preserve the underlying structure of the evaluation space by identifying entities that are undominated under componentwise preference relations. As in Pareto Front let *D* = {*x*^(1)^, …, *x*^(*n*)^}⊂ℝ^*d*^ be a dataset of *d*-dimensional performance vectors (e.g., for universities: publications, citations, internationalization, teaching proxies). Assuming that all dimensions have been harmonized so that higher values are preferable, the standard Pareto dominance relation is formalized together with the corresponding skyline set in Equations 25 and 26:

For two points *x, y* ∈ *D* we say that *x* *dominates* *y* (denoted *x*≻*y*) if and only if


$$\begin{array}{l} x_{j}\geq y_{j}\forall j\in \left\{1,\ldots ,d\right\}\;\text{and }\exists k:x_{k}>y_{k} \end{array}$$ (25)

The skyline set (or Pareto-optimal set) is then given by


$$\begin{array}{l} \text{Sky}\left(D\right)=\left\{x\in D:∄y\in D\text{ such that }y≻x\right\} \end{array}$$ (26)

Equivalently, Sky(*D*) is the first Pareto front. From an order-theoretic perspective, the skyline operator does not produce a scalar score or a total order, but rather identifies the set of maximal elements of the partial-order filter (*D*, ≻). In database research, this operator was formalized as a query primitive by (Borzsonyi et al. 2001).

In evaluation contexts, skyline computation provides a weight-free alternative to composite ranking systems, since it does not impose explicit trade-offs among criteria and avoids collapsing heterogeneous criteria into a single composite indicator.

Skyline techniques have recently been transferred to scientometric analysis and university ranking (Sidiropoulos et al., 2016; Stoupas et al., 2020, 2021). A significant part of research worldwide has focused on extending Skyline methods with secondary ranking mechanisms such as dominance depth, scoring within the Skyline, or representative selection, aiming to improve decision support while maintaining the non-weighted and preference-light philosophy of the Skyline framework.

#### 3.2.1 Why skyline outputs require further discrimination

Skyline operators yield a set of mutually non-dominated points rather than a single, fully ordered ranking. In high-dimensional indicator spaces with weakly correlated attributes, skyline sets can become very large, which makes the output difficult to use in decision-making contexts that require prioritization or systematic comparison among top-performing entities. Consequently, purely set-valued skyline results are often insufficient when stakeholders need a disciplined way to discriminate within the Pareto-optimal region.

To address this limitation, recent research has moved beyond skyline set extraction toward ranking procedures that impose internal structure on skyline points while still avoiding externally imposed weighted aggregation. The objective of skyline ranking is to refine skyline outcomes in a manner that preserves minimal preference assumptions, supports replicability, and reduces the arbitrariness associated with conventional weighted composite scores (Borzsonyi et al., 2001; Sidiropoulos et al., 2016; Stoupas et al., 2020, 2021). In this sense, skyline ranking can be interpreted as a family of post-processing mechanisms that increase resolution without abandoning the non-compensatory semantics of dominance.

Conceptually, most skyline ranking approaches can be classified into four broad families. First, dominance-based scoring schemes quantify the extent to which a skyline point dominates the remainder of the dataset, often discounting dominated objects that are themselves easy to dominate. Second, depth-based or layer-based refinements exploit Pareto depth (iterated skyline layers) to evaluate the robustness of a point's non-dominance and to induce a coarse hierarchy among candidates. Third, graph-based and propagation-based techniques represent dominance relations as networks and rank skyline points by centrality-like notions derived from these dominance-induced structures. Fourth, geometric or ideal-point proximity methods rank skyline points by their closeness to an empirically defined reference (e.g., an ideal vector), thereby providing a continuous ordering without committing to explicit trade-off weights.

Taken together, these approaches form a continuum. At one end lies pure skyline computation, which returns the unordered set Sky(*D*) of non-dominated points. A middle position is occupied by skyline layering, which returns successive skyline layers and thereby induces a partial order through Pareto depth. At the other end lie skyline ranking methods, which apply secondary ordering principles within (and sometimes across) skyline layers, increasing discrimination while avoiding a return to fixed-weight composite scoring.

Across these variants, discrimination is enhanced through dominance structure and principled criteria such as graph centrality, information-retrieval inspired scoring, or multi-criteria ideality, while retaining the transparency and non-compensatory character of dominance-based evaluation. The overarching methodological challenge is therefore to increase interpretability and resolution without reintroducing the normative fragility of arbitrary linear weighting.

#### 3.2.2 Dominance depth and Skyline layering (Pareto depth)

A common extension is *skyline layering* (or Pareto depth). Let *L*_1_ = Sky(*D*) be the first skyline layer and subsequent layers are defined recursively. Equations 27 and 28 formalize skyline layering and the associated dominance-hierarchy score.


$$\begin{array}{l} L_{t}=\text{Sky}\left(D\backslash \underset{r<t}{⋃}L_{r}\right),\;t=2,3,\ldots \end{array}$$ (27)

Each object *x* ∈ *D* is assigned a depth *t*(*x*), corresponding to the layer in which it appears. The index *t* is the *dominance depth* (or Pareto front number). Layering induces a coarse ranking by partial order rather than a strict total ranking, where points in lower-index layers are “better” in the sense of dominance.

Layering yields a tiered classification scheme, by partial order rather than strict total ranking, where institutions in lower-index layers are “better” in the sense of dominance, but within-layer points remain incomparable. This tiered perspective aligns naturally with the argument that multi-dimensional evaluation should be interpreted in equivalence classes rather than strict absolute orders (Borzsonyi et al., 2001; Stoupas and Sidiropoulos, 2021).

Dominance-depth (layer) structure can be incorporated directly into scoring. If *y* lies in layer *L*_*k*_, then dominating *y* may be considered stronger evidence than dominating a deep-layer point. A dominance-hierarchy weighted score is:


$$\begin{array}{l} S_{\text{hier}}\left(x\right)=\underset{y:x≻y}{\sum }w\left(\text{depth}\left(y\right)\right) \end{array}$$ (28)

with *w*(*k*) decreasing in *k*, so that dominating higher-quality (shallower-layer) points contributes more than dominating deep-layer points. Depth-based schemes also support lexicographic ranking rules of the form (*t*(*x*), ϕ(*x*)), where *t*(*x*) is the skyline depth and ϕ(*x*) is a tie-breaking criterion derived from dominance structure. This approach naturally produces tiers of excellence instead of a possibly misleading single order, a property that is particularly attractive in university ranking contexts.

### 3.3 Extensions of skyline

#### 3.3.1 Top-k selection from multilevel skylines

Many applications require a bounded output size, so skyline research has developed methods that produce top-k representatives from multi-level skyline structures. Equation 29 gives the corresponding top-k selection rule over the multilevel skyline candidate pool. A standard top-k approach is:

1. compute Skyline layers *L*_1_, *L*_2_, …  until enough candidates exist
2. apply a dominance or geometry scoring function *S*(·) to rank candidates within layers
3. return the top-*k* candidates.

If $C_{m}=\cup_{i=1}^{m}L_{i}$ is the candidate pool, then select


$$\begin{array}{l} \text{Top}k=\underset{k}{arg top}\left\{S\left(x\right):x\in C_{m}\right\} \end{array}$$ (29)

where *S* is one of the Skyline-ranking scores above. The crucial methodological question is how to define *S*(·) so as to improve discrimination while retaining dominance-based semantics.

#### 3.3.2 Dominance-based scoring of skyline points: dp–idp

A prominent refinement family quantifies how strongly a skyline point dominates the remainder of the dataset (Borzsonyi et al., 2001; Martin-Nevot et al., 2025). Equations 30–33 define the dominated-set notation and the dp-idp scoring mechanism used to discriminate among skyline points. Let


$$\begin{array}{l} \Gamma \left(s\right)=\left\{p\in D\backslash \text{Sky}\left(D\right):s≻p\right\} \end{array}$$ (30)

denote the set of non-skyline points dominated by a skyline point *s*. Naïve scoring schemes based on |Γ(*s*)| are insufficient, as they treat all dominated points as equally informative. Modern discriminative approaches assign higher value to dominating points that are difficult to dominate.

#### 3.3.3 Dominance power–inverse dominance power scoring

Inspired by the tf–idf (term frequency-inverse document frequency) paradigm in information retrieval, the *dp–idp* weighting scheme defines an inverse dominance power for each dominated point *p* as


$$\begin{array}{l} \text{idp}\left(p\right)=\log\frac{\left|\text{Sky}\left(D\right)\right|}{\left|\left\{s\in \text{Sky}\left(D\right):s≻p\right\}\right|} \end{array}$$ (31)

which downweights points dominated by many skyline elements. A local contribution of *p* to a specific skyline point *s* via dominance layers based on dominance hierarchy is defined as


$$\begin{array}{l} \text{dp}\left(p,s\right)=\frac{1}{\text{lm}\left(p,s\right)} \end{array}$$ (32)

where lm(*p, s*) denotes a layer-based index reflecting how directly *s* dominates *p*. The resulting overall score of a skyline point *s* is then


$$\begin{array}{l} {\text{Score}}_{\text{dp–idp}}\left(s\right)=\underset{p\in \Gamma \left(s\right)}{\sum }\text{dp}\left(p,s\right)\cdot \text{idp}\left(p\right) \end{array}$$ (33)

This approach promotes skyline points that dominate genuinely competitive points (not points dominated by almost everyone), thereby giving a mathematically grounded “importance” notion without introducing institutional weights.

Recent research reports that dp–idp may suffer from ties and efficiency limitations and proposes hierarchy-based improvements to enhance better discrimination among skyline points when the skyline is large (Martin-Nevot et al., 2025).

#### 3.3.4 Graph-based Skyline ranking: RankSky

Another prominent direction models dominance relations among skyline points as a stochastic graph and ranks them using eigenvector-based centrality measures. In RankSky, skyline points form the nodes of a directed graph, with transition probabilities derived from dominance relationships and overlap in dominated regions. Equation 34 gives the PageRank-style stationary equation used in the RankSky formulation. The ranking vector *r* is computed as the stationary solution of a PageRank-style equation:


$$\begin{array}{l} r=\alpha P^{⊤}r+\left(1-\alpha \right)v \end{array}$$ (34)

where *P* is a row-stochastic transition matrix, *v* is a teleportation vector (typically uniform), and α ∈ (0, 1) is a damping factor. The resulting eigenvector ranks skyline points by their structural importance within the dominance graph. The objective is analogous to PageRank: a skyline point is important if it is “endorsed” by the dominance structure in a principled, convergent linear-algebraic way, while remaining independent of externally specified criterion weights (Martin-Nevot et al., 2025).

#### 3.3.5 MCDA-inspired skyline ranking: CoSky

Multi-Criteria Decision Analysis (MCDA) denotes a family of formal methods for evaluating a finite set of alternatives under multiple, potentially conflicting criteria. Equations 35–39 give the candidate-vector representation, ideal-point construction, normalization step, and CoSky similarity score. Let the set of candidates be *A* = {*a*_1_, …, *a*_*n*_} and let each alternative *a*_*i*_ be represented by a performance vector


$$\begin{array}{l} x_{i}=\left(x_{i1},x_{i2},\ldots ,x_{id}\right)\in {\mathbb{R}}^{d} \end{array}$$ (35)

where *x*_*ij*_ measures performance on criterion *j*. MCDA methods address the fact that no single criterion is sufficient, and that trade-offs between criteria must be handled in a principled way.

A widely used MCDA principle is TOPSIS (Technique for Order Preference by Similarity to the Ideal Solution), which ranks alternatives by proximity to an *ideal* solution and remoteness from an *anti-ideal* solution. After an appropriate normalization and, in classical TOPSIS, optional weighting, one defines:


$$\begin{array}{l} I^{+}=\left(\underset{i}{\max}x_{i1}^{\prime },\underset{i}{\max}x_{i2}^{\prime },\ldots ,\underset{i}{\max}x_{id}^{\prime }\right),\\ \;I^{-}=\left(\underset{i}{\min}x_{i1}^{\prime },\underset{i}{\min}x_{i2}^{\prime },\ldots ,\underset{i}{\min}x_{id}^{\prime }\right) \end{array}$$ (36)

where $x_{ij}^{\prime }$ denotes the normalized performance of alternative *i* on criterion *j*. In its standard form, TOPSIS computes distances from *I*^+^ and *I*^−^ (often Euclidean) and uses a relative-closeness score to induce an ordering. The conceptual core is that a strong alternative should be simultaneously close to the best attainable profile and far from the worst profile.

CoSky transfers ideal-point reasoning into skyline analytics. As a skyline-point ranking method proceeds in two stages: (i) compute the skyline set to retain only Pareto-optimal candidates, and (ii) rank the resulting skyline points using a continuous similarity-to-ideal score. The first step ensures that all ranked objects are non-dominated under Pareto semantics, preserving the multi-criteria integrity of the evaluation. The second step provides discrimination among skyline points without reverting to externally specified trade-off weights.

Given the original dataset *D* = {*x*_1_, …, *x*_*n*_}, CoSky uses a normalization such as:


$$\begin{array}{l} x_{ij}^{\prime }=\frac{x_{ij}}{\sum_{i=1}^{n}x_{ij}} \end{array}$$ (37)

which produces comparable scale across criteria. Unlike conventional composite rankings, CoSky avoids manually assigned weights and instead derives attribute weights using dispersion measures such as the Gini index, so that dimensions with greater inequality (and thus higher discriminative power) contribute more strongly to the final score (Alouaoui et al., 2019; Martin-Nevot et al., 2025).

After normalization, an ideal point *I*^+^ is constructed component-wise from the best observed normalized values over the candidate set:


$$\begin{array}{l} I^{+}=\left(\underset{i}{\max}x_{i1}^{\prime },\underset{i}{\max}x_{i2}^{\prime },\ldots ,\underset{i}{\max}x_{id}^{\prime }\right) \end{array}$$ (38)

CoSky then assigns a score to each skyline point by measuring its proximity to *I*^+^ using Salton's cosine similarity. Writing $x_{i}^{\prime }=\left(x_{i1}^{\prime },\ldots ,x_{id}^{\prime }\right)$, the CoSky score is:


$$\begin{array}{l} {\text{Score}}_{\text{CoSky}}\left(i\right)=\cos\left(x_{i}^{\prime },I^{+}\right)=\frac{x_{i}^{\prime }\cdot I^{+}}{||x_{i}^{\prime }||||I^{+}||} \end{array}$$ (39)

Conceptually, CoSky yields a continuous ranking of skyline points according to how closely their normalized performance vectors align with an ideal performance vector constructed from the data itself. In contrast to fixed-weight composite systems, the method retains skyline admissibility as a first-stage guarantee of non-domination and then applies a transparent, reproducible scoring rule for within-skyline discrimination (Martin-Nevot et al., 2025).

#### 3.3.6 DeepSky and multilevel discrimination

To address both (i) large skylines and (ii) the need for finer discrimination, recent work proposes DeepSky, which combines multilevel skyline decomposition with one of the skyline-point scoring schemes (dp-idp/RankSky/CoSky). The dataset is first partitioned into skyline layers, after which a ranking operator is applied within and across layers to return a controllable, interpretable output of the top-*k* “best” skyline points. This approach balances interpretability with computational efficiency in large datasets while preserving the dominance-based foundation (Alouaoui et al., 2019; Martin-Nevot et al., 2025).

#### 3.3.7 Relaxed dominance: *k*-dominance and partial dominance

A widely studied relaxation of strict Pareto dominance requires superiority in only a subset of dimensions rather than across all criteria (Chan et al., 2006). In this setting, dominance is imposed in at least *k* dimensions, where *k*<*d*. Equation 40 defines the k-dominance relation used to relax strict Pareto dominance. Formally, for two performance vectors, *x* is said to *k-dominate* *y* if


$$\begin{array}{l} x{≻}_{k}y\;\Leftrightarrow \;\left|\left\{j:x_{j}\geq y_{j}\right\}\right|\geq k\text{ and }\exists j\text{ such that }x_{j}>y_{j} \end{array}$$ (40)

By weakening the dominance condition, *k*-dominance significantly reduces the size of skyline-like result sets, especially in high-dimensional spaces where strict Pareto skylines tend to grow rapidly. This relaxation improves both computational tractability and interpretability, while preserving the core dominance-based semantics of skyline evaluation.

#### 3.3.8 Flexible skylines via monotone function families

Beyond fixed cardinality constraints, more general forms of partial dominance have been proposed through *flexible skyline* models extending classical skyline semantics. In these approaches, dominance is defined relative to a family of monotone aggregation functions rather than componentwise comparison alone (Ciaccia and Martinenghi, 2020). An object is considered preferable to another if it outperforms it under all functions in the family, thereby ensuring robustness across a range of plausible preference models. This framework accommodates uncertainty or variability in decision-maker priorities without committing to explicit weights, extending the dominance paradigm to preference-robust evaluation while maintaining its non-compensatory character. Equation 41 defines flexible dominance with respect to a family of monotone aggregation functions.

Let F denote a set of monotone functions *f*:ℝ^*d*^ → ℝ. For two performance vectors *x, y* ∈ *X*, flexible dominance is defined as


$$\begin{array}{l} x{≻}_{F}y\;\Leftrightarrow \;\left(\forall f\in F\right)f\left(x\right)\geq f\left(y\right)\text{ and }\left(\exists f\in F\right)f\left(x\right)>f\left(y\right) \end{array}$$ (41)

Under this definition, an object dominates another if it performs at least as well under every admissible aggregation function in F, and strictly better under at least one. This formulation generalizes Pareto dominance and accommodates a broader range of admissible preference models while remaining grounded in order-theoretic principles.

The resulting F-skyline captures *preference robustness*, identifying entities that are optimal across an entire family of plausible monotone preference functions rather than a single weight vector. By avoiding commitment to a specific aggregation rule, flexible skylines provide a principled way to incorporate uncertainty or heterogeneity in decision-maker preferences without reverting to explicit weighting. In this sense, flexible skyline models bridge the gap between classical skyline querying and preference-aware ranking, offering enhanced expressiveness while preserving the non-compensatory and weight-free character that distinguishes dominance-based evaluation from conventional composite ranking systems.

#### 3.3.9 Regret minimization and k-regret sets

Regret-minimization techniques address the challenge of preference uncertainty by selecting a small representative subset *R*⊆*D* without committing to a single scoring or aggregation function. Rather than assuming a fixed utility model, these methods consider a family of admissible utility functions U, typically monotone functions over the evaluation criteria (Nanongkai et al., 2010). Equations 42 and 43 define the regret ratio and the associated k-regret minimization objective. For a given utility function *u* ∈ U, the *regret ratio* of a representative set *R* is defined as


$$\begin{array}{l} \text{rr}\left(R,u\right)=1-\frac{\max_{x\in R}u\left(x\right)}{\max_{x\in D}u\left(x\right)} \end{array}$$ (42)

which quantifies the relative loss incurred when selecting the best element from *R* instead of from the full dataset *D*. A regret ratio of zero indicates that *R* contains an optimal solution for the utility function *u*, whereas larger values indicate increasing loss.

A *k-regret query* seeks a subset *R* of cardinality *k* that minimizes the worst-case regret over all admissible utility functions (Nanongkai et al., 2010; Chester et al., 2014), that is,


$$\begin{array}{l} \underset{|R|=k}{\min}\underset{u\in U}{\max}\text{rr}\left(R,u\right) \end{array}$$ (43)

This formulation yields a compact, size-controlled output that is robust to unknown or varying preferences. Regret-minimization methods are closely related to skyline semantics: skyline points typically incur zero regret for at least one utility function, and regret-based representatives can be interpreted as approximations of the skyline that trade completeness for succinctness. As such, *k*-regret sets provide a principled compromise between pure skyline outputs and fully ordered rankings, preserving preference robustness while ensuring practical usability in decision-support settings.

### 3.4 Rainbow ranking and the RR-index

Rainbow ranking is a skyline-inspired methodology that preserves the multi-criteria semantics of performance evaluation by relying on Pareto (componentwise) dominance, avoiding score aggregation via weighted sums (Stoupas et al., 2018; Sidiropoulos et al., 2017). Rather than producing a total order, Rainbow Ranking assigns each entity a categorical profile, mapping each performance dimension to a discrete level and producing a vector of categories. The central idea is to partition entities into *equivalence classes* induced by iterative skyline extraction (non-dominated sorting), and then map the resulting tier structure to a normalized scalar score, the *RR-index*, which remains interpretable while avoiding explicit trade-off weights (Stoupas et al., 2018, 2021).

#### 3.4.1 Dominance and skyline

As previously in the skyline method definition, let $A=\left\{x_{1},\ldots ,x_{n}\right\}\subset {\mathbb{R}}^{d}$ be a set of entities (e.g., universities) represented by *d* indicators, oriented so that larger values are preferred. The dominance relation and the corresponding skyline set used in Rainbow Ranking are restated in Equations 44 and 45. Define Pareto dominance:


$$\begin{array}{l} x≻y\Leftrightarrow \left(\forall j\in \left\{1,\ldots ,d\right\}:x_{j}\geq y_{j}\right)\;\wedge \;\left(\exists j:x_{j}>y_{j}\right). \end{array}$$ (44)

The (first) skyline set is then:


$$\begin{array}{l} \text{Sky}\left(A\right)=\left\{x\in A:∄y\in A\text{ such that }y≻x\right\}. \end{array}$$ (45)

Because ≻ is a strict partial order, Sky(*A*) is generally an antichain, implying incomparability among skyline members and motivating structured post-processing for decision support (Chester and Assent, 2015).

#### 3.4.2 Rainbow layers (iterative skylines)

Rainbow Ranking constructs ordered layers (*S*_1_, *S*_2_, …, *S*_*K*_) by repeatedly extracting the skyline. Equation 46 formalizes the iterative extraction of Rainbow Ranking layers:


$$\begin{array}{l} \begin{array}{c} A^{\left(1\right)}=A,\;S_{1}=\text{Sky}\left(A^{\left(1\right)}\right),\\ A^{\left(k+1\right)}=A^{\left(k\right)}\backslash S_{k},\;S_{k+1}=\text{Sky}\left(A^{\left(k+1\right)}\right) \end{array} \end{array}$$ (46)

until *A*^(*K*+1)^ = ∅. Each *S*_*k*_ is interpreted as an equivalence class under non-dominance, yielding a tiered evaluation that avoids forcing a strict total order (Stoupas et al., 2018; Sidiropoulos et al., 2017).

#### 3.4.3 RR-index (scalar summary without weighted sums)

To obtain an interpretable scalar while preserving the layer semantics, Rainbow Ranking defines the RR-index. Equation 47 defines the above-layer and within-layer tie sets used in the RR-index. Let *S*(*x*) denote the skyline layer containing entity *x* (i.e., *x* ∈ *S*_ℓ_ for some ℓ). Define:


$$\begin{array}{l} A_{\text{above}}\left(x\right)=\left\{y\in A:y\in S_{r},\;\forall r<\ell \right\},\;A_{\text{tie}}\left(x\right)=\left\{y\in S_{\ell }:y\neq x\right\} \end{array}$$ (47)

The RR-index is:


$$\begin{array}{l} RR\left(x\right)=100-100\cdot \frac{\left|A_{\text{above}}\left(x\right)|+\frac{1}{2}|A_{\text{tie}}\left(x\right)\right|}{\left|A\right|},\;0<RR\left(x\right)\leq 100. \end{array}$$ (48)

This approach aligns with Moed's critique (Moed, 2017) that global rankings should not collapse diverse missions into a single metric. Rainbow ranking has been applied to over 105,000 scientists (Stoupas et al., 2018) and to national-level evaluations of academic departments (Sidiropoulos et al., 2017), demonstrating its scalability and interpretability. Thus, *RR*(*x*) is determined by the dominance-induced stratification of the dataset rather than compensatory weighting of indicators (Stoupas et al., 2018, 2021).

Recent extensions adjust the underlying partial order to increase discrimination while maintaining the equivalence-class philosophy, including majorization-based variants that replace Pareto dominance with a majorization relation over suitably normalized vectors (Stoupas and Sidiropoulos, 2021). The complete Rainbow Ranking and RR-index computation is summarized in Algorithm 1.

Algorithm 1 **Rainbow ranking and RR-index computation**

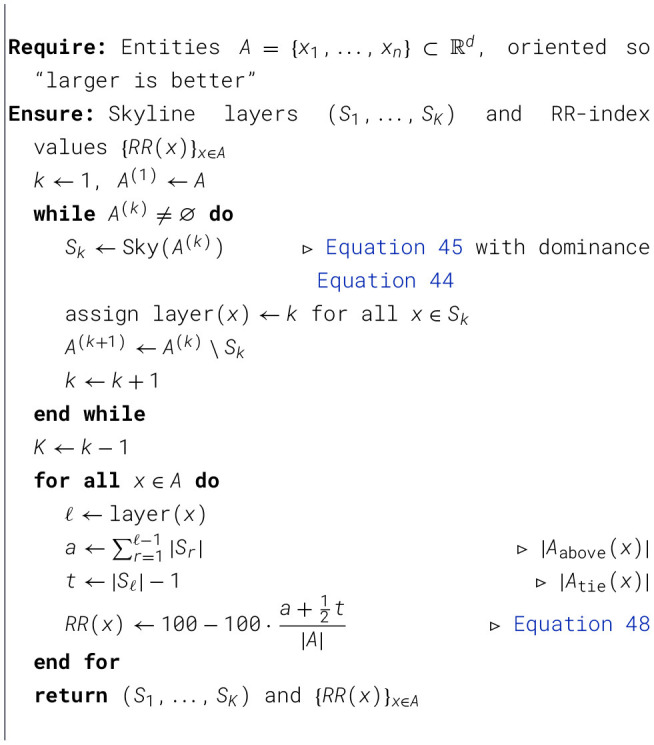

### 3.5 Conclusion—Methodological implications of dominance-based methods

Over the last decade, dominance-based evaluation has matured from a set-valued notion of Pareto efficiency into a coherent family of ranking methodologies that improve discrimination while preserving the multidimensional semantics of non-compensatory comparison. At its core, the *Skyline operator*, introduced by Börzsönyi, Kossmann, and Stocker (Borzsonyi et al., 2001), extracts the set of non-dominated points in multidimensional data—mathematically equivalent to the first Pareto front. By construction, skyline-based ranking provides a mathematically grounded alternative to weighted composite indicators, replacing global trade-off assumptions with partial-order reasoning rather than enforcing a single scalar score.

This paradigm has been successfully transferred to research evaluation. Skyline ranking has been increasingly employed as a methodological framework for shortlisting entities whose performance cannot be surpassed across multiple evaluation criteria. In scientometrics, skyline formulations model each researcher as a point in a multidimensional index space, such as combinations of citation-based indicators, *h*-index variants, or productivity measures, and identify those researchers who are not dominated across multiple evaluation criteria. This approach was systematically introduced to research evaluation by Sidiropoulos et al. (2016), who demonstrated that skyline reasoning provides a natural and mathematically grounded solution to multi-dimensional ranking problems where no single indicator can capture overall performance.

Building on this foundation, skyline-based evaluation has been extended to the domain of university rankings. Stoupas et al. (2021) proposed applying skyline methods to institutions evaluated in indicator spaces without aggregating criteria through fixed or subjective weights. In this setting, universities are compared directly on their performance profiles, and ranking outputs take the form of Pareto fronts or equivalence classes of mutually non-dominated institutions rather than a forced total order. This approach emphasizes transparency, reproducibility, and parameter-free evaluation when grounded in verifiable research output, and avoids reliance on reputation surveys or self-reported data. An empirical assessment of this framework using Microsoft Academic data is reported in Stoupas et al. (2020), illustrating its applicability at scale.

A central advantage of skyline-based methods is that dominance relations remain explicit and auditable, results are reproducible given the data and dominance definition, and preference assumptions are minimized because no single utility function or weight vector must be fixed. At the same time, practical limitations, including skyline cardinality, incomparability among non-dominated points, and computational costs in high dimensions, have motivated substantial methodological refinement. Recent developments such as dp-idp, RankSky, and CoSky (Martin-Nevot et al., 2025) improve discrimination among skyline points by introducing principled secondary ordering mechanisms, including IR-inspired dominance weighting, PageRank-like stochastic propagation, cosine similarity to ideal vectors, and dispersion-based (e.g., Gini) attribute weighting. Overall, modern skyline ranking is best understood as a spectrum ranging from pure Pareto optimality (maximal transparency and minimal preference) to ranked and size-controlled outputs that introduce limited structure (depth, dominance influence, or geometry) while still avoiding the normative fixed-weight paradigm characteristic of conventional composite ranking systems.

Research over the last decade has transformed skyline processing from a purely set-valued query into a rich family of ranking methodologies. Dominance-depth ordering, dominance-based scoring, graph centrality, MCDA-inspired similarity measures, flexible dominance models, and regret-based selection all aim to enhance discrimination while preserving the multidimensional semantics of skyline evaluation. These developments position skyline ranking as a mathematically grounded and conceptually transparent alternative to traditional weighted composite ranking systems.

Finally, skyline-derived categorical models such as Rainbow Ranking and the RR-index (Stoupas et al., 2018; Sidiropoulos et al., 2017), illustrate an important methodological shift: rather than insisting on a strict total order, they stratify entities into ordered equivalence classes and then provide an interpretable scalar summary based on dominance-induced position, not compensatory aggregation. Related extensions that modify the underlying dominance relation, such as majorization-based evaluation (Stoupas and Sidiropoulos, 2021), further reinforce the central implication of this family: dominance-based methods provide transparent, auditable, and reproducible alternatives to weighted composite rankings, producing rankings as structured partial orders (fronts, layers, representatives, or dominance-informed scores) whose meaning remains anchored in multi-criteria comparability rather than in externally imposed trade-offs.

Overall, modern skyline ranking and dominance-based methods can be understood as a spectrum: from pure Pareto optimality (maximal transparency, minimal preference) to ranked and size-controlled outputs that introduce limited structure (depth, dominance influence, or geometry) while still avoiding explicit criterion weighting.

## 4 Non-aggregative profile and tier evaluation frameworks

A prominent class of non-weighted (or minimally weighted) alternatives to global university rank tables replaces scalar composite scores with *multi-dimensional profiles* and *explicit tiering rules*. The central concern motivating these approaches is that traditional rankings typically reduce heterogeneous evidence (e.g., publications, citations, internationalization, teaching proxies, income measures) to a single number through fixed weights, thereby enforcing compensatory trade-offs that are rarely justified as universally valid. By contrast, profile-and-tier frameworks aim to preserve the multi-criteria nature of institutional performance and to communicate results in ways that remain interpretable, transparent, and robust to small measurement fluctuations (European Union, 2015; European Commission, 2014).

Within this family, two design philosophies are most visible in practice. First, *dashboard/profile systems* prioritize *vector-valued* reporting: performance is displayed across multiple dimensions without collapsing them into a global total order. Second, *tier/threshold systems* operationalize evaluation through explicit decision rules that map indicator values to categorical labels (e.g., A–E, top/middle/bottom, or eligibility sets). Importantly, these are not mutually exclusive. Many dashboard systems employ tiering as a presentation layer, precisely because categories reduce the risk that users interpret marginal numeric differences as meaningful ranking gaps. A third, closely related development incorporates *uncertainty-aware tiering*, which uses statistical intervals or significance logic to classify institutions only when differences are robust rather than plausibly attributable to noise (CWTS Leiden Ranking, 2025b; Abramo et al., 2016).

### 4.1 Dashboard/profile systems: vector-valued evaluation without composite ranks

Dashboard-based systems represent each university *i* by a performance vector across *d* indicators. Equations 49–51 formalize the vector-valued dashboard representation, indicator normalization, and user-selected partial comparison logic used in multidimensional reporting:


$$\begin{array}{l} x_{i}=\left(x_{i1},x_{i2},\ldots ,x_{id}\right)\in {\mathbb{R}}^{d} \end{array}$$ (49)

where indicators capture distinct dimensions (e.g., research impact, teaching proxies, international orientation, knowledge transfer). Since indicators differ in scale, direction, and distribution, dashboard systems typically perform direction harmonization and normalization. Let *g*_*j*_(·) align directionality (so larger values mean better performance), and let ϕ_*j*_(·) normalize to a comparable scale:


$$\begin{array}{l} z_{ij}=\phi_{j}\left(g_{j}\left(x_{ij}\right)\right) \end{array}$$ (50)

The key methodological point, however, is that the outcome remains *non-aggregative*: users are encouraged to examine performance patterns across dimensions rather than rely on a single score (European Union, 2015; Moed, 2017). Dashboards *do not* define a fixed-weight composite such as $s_{i}=\underset{j}{\sum }w_{j}z_{ij}$. Instead, they publish the vector *z*_*i*_ (or a categorized version of it), allowing interpretation across dimensions (European Union, 2015; Moed and Halevi, 2015).

A natural formalization of dashboard comparison is a user-selected partial order. If a stakeholder selects an indicator subset *J*⊆{1, …, *d*}, then


$$\begin{array}{l} i{≽}_{J}k\;\Leftrightarrow \;\forall j\in J:z_{ij}\geq z_{kj} \end{array}$$ (51)

This design can be interpreted as producing a *partial order* rather than a total order. When stakeholders prioritize different subsets of indicators, the system supports user-driven comparisons among institutions, acknowledging that universities may be incomparable when one outperforms another in some dimensions but not in others. Such an approach aligns with the goal of preserving institutional diversity and discouraging simplistic claims of universal “best” institutions.

#### 4.1.1 Like-with-like comparison via similarity neighborhoods

Profile-based systems frequently encourage comparisons among institutions with similar missions or contexts. Let $m_{i}\in {\mathbb{R}}^{p}$ denote a mission/profile vector (e.g., research intensity, subject mix, regional engagement). Equation 52 formalizes the like-with-like comparison logic through similarity neighborhoods. Similarity can be captured by a distance *d*(·, ·), such as Euclidean distance:


$$\begin{array}{l} d\left(i,k\right)=||m_{i}-m_{k}{||}_{2} \end{array}$$ (52)

and comparisons may be restricted to a neighborhood N_ϵ_(*i*) = {*k*:*d*(*i, k*) ≤ ϵ}, strengthening interpretive validity by avoiding inappropriate cross-mission comparisons (European Union, 2015; Jongbloed, 2014).

### 4.2 Tier/threshold systems

Tier and threshold approaches replace composite rank tables with explicit *classification rules* that map a university's multi-indicator performance profile to a finite set of categories. Equation 53 formalizes this general mapping from a multidimensional performance profile to a finite set of categories. Formally, they define a mapping


$$\begin{array}{l} C:{\mathbb{R}}^{d}\to T \end{array}$$ (53)

where T may represent performance bands such as {*A, B, C, D, E*} or {Top, Middle, Bottom}. The defining characteristic is that *C*(·) is determined by transparent rule sets (thresholds, quantiles, or significance criteria) rather than by a weighted sum. In many implementations, the rules are *non-compensatory*: weak performance on a required criterion cannot be offset by excellence on other criteria unless such substitution is explicitly allowed (DAAD, 2025).

#### 4.2.1 Banding/grouping rules (A–E, top/middle/bottom)

A widely used mechanism is quantile-based banding. For indicator *j*, let *q*_α_(*x*_·*j*_) denote the α-quantile across institutions. Equation 54 defines a five-band quantile-based classification rule for assigning institutions to performance groups. A five-band scheme can be written as


$$C_{j}(i)=\{\begin{array}{ll} A & x_{ij}\geq q_{0.8}(x_{\cdot j}),\\ B & q_{0.6}\leq x_{ij}<q_{0.8},\\ C & q_{0.4}\leq x_{ij}<q_{0.6},\\ D & q_{0.2}\leq x_{ij}<q_{0.4},\\ E & x_{ij}<q_{0.2}. \end{array}$$ (54)

U-Multirank operationalizes a closely related logic by placing institutions into performance groups (A–E) for each indicator instead of computing a single composite rank-table (European Union, 2015). Similarly, CHE-style systems emphasize grouping (often top/middle/bottom) rather than strict rank positions. Public documentation highlights that group membership is intended to capture meaningful differences while avoiding the misleading precision of a full ordinal ordering. Such group-based reporting can be especially appropriate in contexts where measurement error and heterogeneous missions make strict ordering difficult to justify (DAAD, 2025).

#### 4.2.2 Eligibility and conjunctive thresholds

Another common design defines excellence or eligibility by meeting minimum requirements across a selected indicator set *J*. Let τ_*j*_ be a threshold for indicator *j*. Equations 55 and 56 formalize the conjunctive eligibility rule and its tiered extension using multiple threshold vectors. A conjunctive (checklist) rule is


$$\begin{array}{l} \text{Eligible}\left(i\right)=1\left\{x_{ij}\geq \tau_{j}\;\forall j\in J\right\} \end{array}$$ (55)

Tiered variants use multiple threshold vectors τ^(1)^≻τ^(2)^≻⋯  to assign hierarchical categories:


$$C(i)=\{\begin{array}{ll} \text{Tier 1} & x_{ij}\geq \tau_{j}^{\left(1\right)}\;\forall j\in J,\\ \text{Tier 2} & x_{ij}\geq \tau_{j}^{\left(2\right)}\;\forall j\in J,\\ \vdots & \;\\ \text{Tier K} & \text{otherwise}. \end{array}$$ (56)

Such rules are particularly useful for shortlisting and policy eligibility because the decision logic is auditable, although it remains normative in the selection of *J* and the calibration of τ.

#### 4.2.3 Percentile-based excellence thresholds (bibliometric exemplars)

In research evaluation, excellence is often operationalized through field- and year-normalized percentiles. Equations 57 and 58 formalize the PP10 indicator and the corresponding percentile-based excellence threshold. A canonical university-level indicator is the proportion of publications in the top 10% most cited within field and year:


$$\begin{array}{l} {\text{PP}}_{10}\left(i\right)=\frac{1}{N_{i}}\underset{p\in P_{i}}{\sum }1\left\{p\in \text{Top}\text{-}10\%\left(f\left(p\right),y\left(p\right)\right)\right\} \end{array}$$ (57)

where P_*i*_ is the publication set of institution *i* and the top-10% condition is computed within each field/year stratum. A threshold rule then defines excellence as


$$\begin{array}{l} C\left(i\right)=\text{Excellent }\text{if }{\text{PP}}_{10}\left(i\right)\geq \tau \end{array}$$ (58)

This approach aligns with indicator-level benchmarking systems and is supported by the Leiden Ranking documentation and its associated guidance for interpretation (CWTS Leiden Ranking, 2025a).

### 4.3 Uncertainty-aware tiering (robust classification)

A key critique of deterministic ranks is that many institutions are statistically indistinguishable once uncertainty is acknowledged. Uncertainty-aware tiering addresses this by classifying institutions only when evidence supports clear separation. Equations 59 and 60 formalize the lower-confidence-bound rule and the funnel-plot-based three-tier classification rule used for uncertainty-aware tiering. One approach requires that a lower confidence bound exceed a threshold:


$$\begin{array}{l} C\left(i\right)=\text{Excellent only if }\text{LB}\left({\hat{x}}_{ij}\right)\geq \tau \end{array}$$ (59)

where LB(·) is a reported lower bound (e.g., from bootstrap or stability-interval methods). The Leiden Ranking explicitly supports uncertainty-sensitive interpretation through stability intervals for certain indicators, encouraging caution against over-interpreting marginal differences (CWTS Leiden Ranking, 2025a).

A complementary approach uses funnel-plot logic to classify outliers relative to a benchmark. Let μ_*j*_ be a benchmark (e.g., system mean), ${\hat{x}}_{ij}$ an estimate, and se_*ij*_ its standard error. A three-tier rule can be written as


$$C_{j}(i)=\{\begin{array}{ll} \text{Top} & {\hat{x}}_{ij}-z_{\alpha /2}{\text{se}}_{ij}>\mu_{j},\\ \text{Bottom} & {\hat{x}}_{ij}+z_{\alpha /2}{\text{se}}_{ij}<\mu_{j},\\ \text{Middle} & \text{otherwise}. \end{array}$$ (60)

This type of classification supports “robust tiering” rather than fragile fine-grained ranking and has been advocated to prevent spurious ordering when uncertainty is non-negligible (Abramo et al., 2018).

### 4.4 Strengths and limitations

Non-aggregative tier/threshold frameworks offer several advantages over fixed-weight composite rankings. First, they reduce normative arbitrariness by avoiding a single universal set of compensatory weights, thereby preserving the multidimensional structure of institutional performance profiles (Moed and Halevi, 2015). Second, tiering and banding reduce false precision, making results more interpretable and less sensitive to negligible metric differences (a central rationale of grouping approaches) (DAAD, 2025). Third, uncertainty-aware tiering further improves inferential validity by distinguishing robust differences from noise and sampling variability (CWTS Leiden Ranking, 2025a; Abramo et al., 2018).

Related dashboard-style and group based multidimensional systems including U-Multirank, CHE-style group reporting, and the CWTS Leiden Ranking, have been introduced and discussed in a previous section, where their indicator sets and reporting frameworks are presented in detail. Here, they serve as reference examples for highlighting the broader methodological trade-offs of non-aggregative evaluation.

At the same time, non-aggregative frameworks introduce trade-offs. They do not deliver a single definitive ordering, which may frustrate stakeholders seeking a simple rank table. Moreover, methodological choices in normalization, missing-data handling, and grouping thresholds still affect interpretive outcomes, even if explicit weights are avoided. Consequently, dashboard rankings should be interpreted as *vector-valued transparency instruments* rather than interchangeable substitutes for scalar composite rankings.

Tier and threshold methods convert continuous indicator evidence into categorical outputs such as bands, groups, and eligibility sets. Instead of asking “what is the best university overall?”, these systems typically ask whether an institution meets a defined standard, or whether it belongs in a top group for a given dimension. The mapping from evidence to outcome is given by explicit rules, which makes the approach transparent and auditable. In many cases, the rules are *non-compensatory*: failure on a required criterion cannot be offset by excellence elsewhere unless the evaluation framework explicitly permits such substitution.

Table 4 summarizes the main non-aggregative subfamilies discussed in this section, highlighting the corresponding output types, rule/logic mechanisms, and typical use cases.

**Table 4:** Non-aggregative profile-and-tier evaluation frameworks.

Subfamily	Output type	Rule/logic type	Typical use cases
Dashboard/profile systems	multi-dimensional profile, no global composite rank	normalization for comparability, user-driven comparison	stakeholder-specific exploration, e.g., U-Multirank
Tier/threshold systems	Categorical labels per indicator/ dimension	Explicit decision rules: quantile banding (A–E), conjunctive thresholds	Communication-oriented evaluation, dimension-specific excellence labeling e.g., CHE grouping
Uncertainty-aware tiering	Robust tiers with uncertainty information, “classify only if clearly separated”	Interval- or significance-based assignment	Avoiding false precision, preventing fake year-to-year rank changes, reliable outliers identification

## 5 Frontier and efficiency-based ranking methods

Frontier (efficiency) methods evaluate universities as *decision making units* (DMUs) relative to an empirically constructed best-practice production frontier. In contrast to composite rankings that aggregate heterogeneous indicators through fixed, a priori weights, DEA and FDH infer performance by comparing how efficiently a university transforms inputs (e.g., staff, funding, infrastructure) into outputs (e.g., graduates, publications, citations, knowledge-transfer outcomes). The central output is an efficiency score together with a frontier (efficient) set and peer benchmarks, yielding a natural tiering (frontier vs. non-frontier) rather than a single global rank-table ordering. As a result, DEA and FDH are frequently described as weight-free or non-normative alternatives to composite ranking systems, although they still rely on explicit modeling assumptions regarding orientation, returns to scale, and variable selection (Emrouznejad and Yang, 2018; Johnes, 2015).

Let $x_{i}\in {\mathbb{R}}_{+}^{m}$ denote the input vector and $y_{i}\in {\mathbb{R}}_{+}^{s}$ the output vector of university *i*∈{1, …, *n*}. Frontier methods define a feasible technology set $T\subseteq {\mathbb{R}}_{+}^{m}\times {\mathbb{R}}_{+}^{s}$ and quantify each university's distance to the boundary of *T*, interpreted as relative technical efficiency.

Let each university *i* = 1, …, *n* be characterized by an input vector $x_{i}\in {\mathbb{R}}_{+}^{m}$ (e.g., academic staff, expenditures) and an output vector $y_{i}\in {\mathbb{R}}_{+}^{s}$ (e.g., graduates, publications, citations). Equation 61 formalizes the feasible production set used in frontier-based efficiency evaluation. Frontier methods define a feasible production set


$$\begin{array}{l} T\subseteq {\mathbb{R}}_{+}^{m}\times {\mathbb{R}}_{+}^{s} \end{array}$$ (61)

and measure the relative efficiency of each unit with respect to the boundary of *T*.

### 5.1 Data Envelopment Analysis (DEA)

DEA is a non-parametric linear-programming approach that constructs a piecewise-linear frontier from observed DMUs. Depending on the evaluation goal, DEA can be formulated as *input-oriented* (minimize inputs for given outputs) or *output-oriented* (maximize outputs for given inputs).

#### 5.1.1 CCR model (constant returns to scale)

In the classic constant-returns-to-scale (CCR) envelopment form, input-oriented efficiency for DMU *o* is obtained by solving. Equations 62 and 63 formalize the input-oriented and output-oriented CCR efficiency models, respectively.


$$\begin{array}{l} \underset{\theta ,\lambda }{\min}\;\theta \;\text{ such that }Y\lambda \geq y_{o},X\lambda \leq \theta x_{o},\lambda \geq 0 \end{array}$$ (62)

where $X\in {\mathbb{R}}_{+}^{m\times n}$ and $Y\in {\mathbb{R}}_{+}^{s\times n}$ collect inputs and outputs across universities, and $\lambda \in {\mathbb{R}}_{+}^{n}$ is the vector of intensity variables. The optimal θ^*^ ∈ (0, 1] is the (input-oriented) technical efficiency score; θ^*^ = 1 indicates frontier efficiency.

The corresponding output-oriented CCR model maximizes an expansion factor φ≥1:


$$\begin{array}{l} \underset{\varphi ,\lambda }{\max}\;\varphi \;\text{ such that }Y\lambda \geq \varphi y_{o},X\lambda \leq x_{o},\lambda \geq 0 \end{array}$$ (63)

#### 5.1.2 BCC model (variable returns to scale)

To account for scale effects commonly observed in higher education systems, the BCC model introduces the convexity constraint. Equations 64 and 65 formalize the BCC convexity constraint and the corresponding scale-efficiency measure.


$$\begin{array}{l} 1^{⊤}\lambda =1 \end{array}$$ (64)

yielding *pure technical efficiency* under variable returns to scale. Scale efficiency is often computed as


$$\begin{array}{l} SE_{o}=\frac{\theta_{o}^{CCR}}{\theta_{o}^{BCC}} \end{array}$$ (65)

#### 5.1.3 DEA multiplier form and endogenous weights

DEA is frequently described as “weight-free” in the sense that it does not impose a single global set of exogenous indicator weights. However, DEA can be expressed in a multiplier form that reveals *endogenous* (DMU-specific) weights. Equation 66 formalizes the output-oriented DEA multiplier form with endogenous weights. For example, an output-oriented ratio form is:


$$\begin{array}{l} \underset{u,v}{\max}\;\frac{u^{⊤}y_{o}}{v^{⊤}x_{o}}\;\text{ such that }\frac{u^{⊤}y_{i}}{v^{⊤}x_{i}}\leq 1\;\forall i,u\geq 0,\;v\geq 0 \end{array}$$ (66)

Although DEA avoids externally imposed weights, this formulation shows that it optimizes endogenous, unit-specific weights consistent with the observed technology (Johnes, 2015). Thus, DEA avoids *predefined* weights, but implicitly generates a set of feasible weights per DMU consistent with the observed technology. The method yields (i) an efficiency score indicating the distance of each university from the frontier, (ii) a set of reference peers, and (iii) a natural tier structure distinguishing frontier institutions from inefficient ones (Wolszczak-Derlacz, 2017; Emrouznejad and Yang, 2018).

### 5.2 Free Disposal Hull (FDH)

The Free Disposal Hull (FDH) model relaxes the convexity assumption of DEA and relies solely on free disposability. As a result, the efficient frontier is defined directly by observed units rather than by convex combinations, producing a staircase-shaped, non-convex frontier (Cooper et al., 2007). Equation 67 formalizes the input-oriented FDH efficiency score. For DMU *o*, the input-oriented FDH efficiency score can be expressed as


$$\begin{array}{l} \theta_{o}^{FDH}=\min\left\{\theta :\exists i\text{ such that }y_{i}\geq y_{o}\text{ and }x_{i}\leq \theta x_{o}\right\} \end{array}$$ (67)

This formulation searches for an observed comparator that weakly dominates the evaluated unit in outputs while requiring no more than a proportional reduction in inputs. FDH has been extended in modern work to richer non-convex production structures and robustness settings.

### 5.3 Advanced DEA and FDH models in university evaluation

Universities are not “single-process” producers; rather, they operate as multi-output, multi-mission organizations in which teaching, research, and third-mission activities coexist and interact. This institutional complexity motivates advanced frontier models that go beyond the standard “black-box” representation of production. In particular, multi-activity and network DEA formulations explicitly represent internal structures and the links between stages (e.g., resources devoted to teaching that also shape research capacity, or research outputs that feed external engagement). By modeling these internal production relationships, network and multi-stage approaches allow efficiency to be decomposed into component efficiencies, making it possible to localize the sources of inefficiency (for example, whether underperformance is primarily associated with teaching processes, research processes, or the interface between them). Such decomposition is especially valuable in university evaluation, where stakeholders often require diagnostically meaningful results rather than a single undifferentiated score.

A recurrent concern in higher-education applications is that DEA/FDH estimators can be sensitive to sampling variation, measurement error, and outliers. To address these issues, many studies employ bootstrap-based procedures to correct bias and to attach measures of statistical precision to efficiency estimates. In practice, bootstrap DEA is frequently embedded in two-stage designs: in the first stage, efficiency scores are estimated; in the second stage, these scores are related to environmental or contextual variables that may influence performance but are not directly controlled by the institution (e.g., funding regimes, student composition, or regional conditions). This combination supports both improved statistical inference and a clearer separation between managerial inefficiency and exogenous constraints, and it has become a common empirical strategy in the higher-education efficiency literature (Agasisti and Johnes, 2015; Simar and Wilson, 2013).

Another methodological challenge arises from the fact that many universities may be assessed as efficient, i.e., they lie on the frontier with θ = 1. When the efficient set is large, efficiency scores alone provide limited discrimination among top-performing institutions. Consequently, a range of ranking and refinement procedures is often used to generate additional differentiation while remaining consistent with the frontier logic. Typical approaches include super-efficiency models (which evaluate a unit relative to a frontier constructed without it), cross-efficiency evaluation (which leverages peer-appraisal across multiple weight vectors), and frontier layering (which iteratively removes efficient units to form successive “tiers” of performance). These methods aim to preserve the benchmarking interpretation of DEA/FDH while enabling more fine-grained comparisons within the efficient subset.

### 5.4 Interpreting outputs and ranking implications

Frontier approaches typically yield (i) a frontier set of efficient universities, (ii) efficiency scores for the remaining institutions, and (iii) peer/reference sets and improvement targets (slacks). This structure naturally supports tier-like interpretation (efficient vs. inefficient, or multi-frontier layering), and rankings among non-frontier institutions can be obtained by ordering efficiency scores. However, because many universities may be efficient (score = 1), additional techniques such as super-efficiency or cross-efficiency are sometimes used to discriminate within the efficient set, analogous to “ranking within the skyline” in Pareto-based methods.

Despite their appeal as alternatives to fixed-weight composites, frontier methods are not assumption-free. Results depend on orientation (input vs. output), returns-to-scale assumptions (CCR vs. BCC), variable selection and dimensionality (too many indicators can inflate efficiency), treatment of missing data, and robustness procedures. Accordingly, DEA/FDH should be interpreted as transparent, model-based benchmarks of relative efficiency rather than direct substitutes for scalar rank-tables (Emrouznejad and Yang, 2018; Johnes, 2015).

## 6 Data-driven reduction (latent-dimension rankings)

Data-driven reduction (latent-dimension) rankings constitute a distinct alternative to designer-specified weighted composite indicators. While these approaches still produce a single scalar score or total order, the effective weights are estimated from the data rather than pre-specified by ranking designers. For this reason, they are sometimes described as “weight-free” or “non-normative”; however, the resulting score remains an implicit aggregation of indicators through statistically inferred loadings. In practice, such methods are used to uncover a small number of latent dimensions underlying observed indicators and to construct thrifty indices reflecting dominant empirical structures in the data (Moed, 2017; Brankovic et al., 2018; Visbal-Cadavid et al., 2020).

### 6.1 Principal component analysis (PCA)-based indices

Let *X* ∈ ℝ^*n*×*d*^ denote the data matrix of *n* universities described by *d* indicators. After harmonization and standardization, PCA identifies orthogonal directions that maximize variance. Equations 68–70 formalize the PCA optimization problem, the equivalent eigenvalue formulation, and the resulting composite ranking score. The first principal component is obtained by solving


$$\begin{array}{l} w_{1}=\arg\underset{||w||=1}{\max}\text{Var}\left(Zw\right) \end{array}$$ (68)

$$\begin{array}{l} \Sigma w_{1}=\lambda_{1}w_{1} \end{array}$$ (69)

where $\Sigma =\frac{1}{n-1}Z^{⊤}Z$ is the sample covariance matrix. A composite ranking score for institution *i* is then


$$\begin{array}{l} s_{i}=z_{i}^{⊤}w_{1} \end{array}$$ (70)

with the entries of *w*_1_ acting as implicit data-driven weights. Extensions include multi-component indices. Equation 71 formalizes the multi-component PCA-based ranking score.


$$\begin{array}{l} s_{i}=\overset{K}{\underset{k=1}{\sum }}\alpha_{k}z_{i}^{⊤}w_{k} \end{array}$$ (71)

where α_*k*_ is often proportional to explained variance, and block-wise PCA to limit dominance by correlated indicator clusters (Muzamhindo et al., 2017; Brankovic et al., 2018).

A frequent application of (robust) PCA method is for diagnostic use in the indicator space of major global rankings (especially ARWU), in order to uncover latent dimensions and to show that a single composite score can conflate distinct latent constructs (e.g., “overall output” vs. “elite top-end excellence”). This is a direct empirical use of PCA to analyze and sometimes reinterpret ranking outcomes (Dehon et al., 2010).

A more recent longitudinal study applies robust PCA and factor analysis across multiple years and ranking systems (e.g., QS/THE/ARWU) to identify stable latent dimensions underlying the published rank tables and to study stability over time (Selten et al., 2020).

Furthermore, beyond diagnosis, there are studies that explicitly use PCA (or modified PCA) to construct an overall performance index and then rank universities based on this index. For example, an analysis that applies a modified PCA framework constructs an overall competitiveness index and produces a derived ranking of Russian universities (Vashchenko et al., 2022).

### 6.2 Factor analysis and latent constructs

Factor-analytic approaches model indicators as noisy manifestations of latent variables. Equation 72 formalizes the factor-analytic measurement model used to represent observed indicators through latent factors:


$$\begin{array}{l} x_{i}=\mu +\Lambda f_{i}+\varepsilon_{i} \end{array}$$ (72)

where Λ ∈ ℝ^*d*×*K*^ is the factor-loading matrix, *f*_*i*_ are latent factor scores, and ε_*i*_ captures idiosyncratic noise. A single-factor specification yields a scalar latent score ${\hat{f}}_{i}$ usable for ranking, while multi-factor models permit dimension-specific ordering or secondary aggregation. Unlike PCA, factor analysis explicitly separates common variance from measurement error, which is advantageous when indicators serve as imperfect proxies for institutional performance (Brankovic et al., 2018; Moed, 2017).

### 6.3 Multi-block latent reduction

University indicators are often organized into thematic blocks (e.g., research, teaching, third mission). Multi-block methods such as Multiple Factor Analysis (MFA) normalize each block before global dimension reduction, preventing large or highly correlated blocks from dominating the latent structure. Let *X*^(*b*)^ denote block *b*; MFA scales each block by its leading singular value before joint analysis. A global latent score can then be derived while preserving balance across dimensions (Visbal-Cadavid et al., 2020).

Multi-block latent reduction has been used in higher-education performance contexts to reduce multi-indicator profiles into a smaller latent space, which can then support composite scoring, clustering, and ranking of institutions while balancing indicator blocks. Dehon et al. (2010) applies MFA in a higher-education evaluation setting and uses the reduced representation for comparative institutional assessment.

### 6.4 Latent variable models and Bayesian approaches

More general latent-variable models treat institutional quality as an unobserved trait θ_*i*_ generating observed indicators. Equations 73 and 74 formalize the continuous-indicator latent-variable model and the binary/ordinal IRT-style model, respectively. For continuous indicators,


$$\begin{array}{l} x_{ij}|\theta_{i}\sim N\left(\alpha_{j}+\beta_{j}\theta_{i},\;\sigma_{j}^{2}\right) \end{array}$$ (73)

where β_*j*_ captures the discriminating power of indicator *j*. Bayesian estimation yields posterior distributions for θ_*i*_, enabling probabilistic rankings and uncertainty-aware comparisons. For binary or ordinal indicators, Item Response Theory (IRT)-style models can be applied:


$$\begin{array}{l} \mathrm{Pr}\left(x_{ij}=1|\theta_{i}\right)={\text{logit}}^{-1}\left(a_{j}\left(\theta_{i}-b_{j}\right)\right) \end{array}$$ (74)

with *a*_*j*_ and *b*_*j*_ denoting discrimination and difficulty parameters, respectively (Heil and Greene, 2023; Guarino et al., 2005).

A typical example comes from Claassen in 2015 (Claassen, 2015), who treats “university quality” as a latent variable and models the observed outcomes/ranking indicators as noisy measurements of this latent characteristic, estimated in a Bayesian hierarchical framework. The result is a posterior distribution over the latent quality of each university, allowing for rankings with uncertainty (credible intervals, posterior probabilities of ranking).

### 6.5 Normalization and identifiability considerations

Despite their data-driven nature, latent-dimension rankings depend on important modeling choices. PCA and factor models are sensitive to scaling and indicator directionality, component signs are not uniquely defined, and factor rotations may alter interpretation. Moreover, estimated loadings depend on the sample of institutions included, implying that rankings may shift when the comparison set changes. Handling missing data also differs across methods, with latent-variable models offering principled solutions under explicit assumptions (Brankovic et al., 2018; Moed, 2017).

### 6.6 Strengths and limitations

Latent-dimension ranking approaches reduce normative arbitrariness by allowing aggregation structures to emerge from empirical covariance patterns rather than from exogenously imposed weights. By applying techniques such as principal component analysis or factor analysis, these methods can uncover the dominant constructs underlying a set of indicators, thereby offering insight into what existing ranking variables primarily measure (for example, reputational standing vs. research performance). Moreover, when implemented through Bayesian or latent-trait models, these approaches can provide uncertainty estimates and probabilistic comparisons, which supports more robust interpretation and aligns naturally with tier-based or uncertainty-aware evaluation frameworks (Moed, 2017; Visbal-Cadavid et al., 2020).

Despite their data-driven nature, latent-dimension rankings do not eliminate weighting but instead replace explicit normative weights with implicit statistical ones. As a result, composite scores may be dominated by the strongest covariance structures in the data, which can reflect size effects, disciplinary composition, or historical prestige rather than a balanced or policy-relevant notion of institutional quality. In addition, maximizing variance does not guarantee conceptual validity, and indicators with high variance may exert disproportionate influence even if their substantive importance is limited. Finally, latent structures are inherently sample-dependent, meaning that rankings derived from them may vary across countries, disciplines, or time periods, thereby complicating cross-system and longitudinal comparability (Brankovic et al., 2018).

## 7 Alternative ranking models—Voting systems and aggregated ranking

Voting systems and aggregated ranking mechanisms constitute a core domain of computational social choice, which studies how individual preferences can be combined into collective decisions using mathematically grounded procedures (Brandt et al., 2016). While classical theories often assume equal voting power among participants, many real-world decision environments, including corporate governance boards, federated systems, and international institutions, feature unequal voting influence. These weighted systems require rigorous mathematical tools to evaluate influence, fairness, and collective rationality (Felsenthal and Machover, 1998; Laruelle and Valenciano, 2008). This section consolidates the main models and algorithms used to (i) quantify influence in weighted collective decisions and (ii) aggregate ranked preferences into collective rankings.

### 7.1 Weighted voting systems

A weighted voting system assigns each voter a non-negative weight representing formal influence. Equations 75 and 76 formalize the weighted voting game and the corresponding winning-coalition condition. A standard representation is the weighted voting game:


$$\begin{array}{l} \left(q;w_{1},w_{2},\ldots ,w_{n}\right) \end{array}$$ (75)

where *w*_*i*_ denotes the weight of voter *i*, and *q* is the quota required for the adoption of a proposal. A coalition *S* is successful if


$$\begin{array}{l} \underset{i\in S}{\sum }w_{i}\geq q \end{array}$$ (76)

Weighted voting systems often display non-linear and counterintuitive relationships between voting weights and actual decision-making power, motivating the use of mathematical power indices (Leech, 2013). Voting weights *w*_*i*_ may differ substantially from a voter's *effective* ability to change outcomes under the quota rule. Therefore, the analysis of weighted voting systems typically proceeds via *power indices*, which quantify each voter's a priori influence by counting criticality or pivot probabilities. Two main canonical measures are Banzhaf and Shapley–Shubik power indices.

#### 7.1.1 Banzhaf power index

After normalization, the Banzhaf index measures the frequency with which a voter is *critical*. It is capable of turning a losing coalition into a winning one. Equation 77 defines the normalized Banzhaf power index. If η_*i*_ denotes the number of coalitions in which voter *i* is critical, the normalized index is:


$$\begin{array}{l} \beta_{i}=\frac{\eta_{i}}{\sum_{j=1}^{n}\eta_{j}} \end{array}$$ (77)

Recent research has focused on efficient computation and approximation for large-scale weighted voting games (Bachrach et al., 2010; Leech, 2003).

#### 7.1.2 Shapley–Shubik power index (SSPI)

The Shapley–Shubik power index interprets power as the probability that a voter is *pivotal* in a uniformly random permutation of voters. Let *v*(·) denote the characteristic function of the voting game (with *v*(*S*) = 1 if *S* is winning and 0 otherwise). Equation 78 defines the Shapley-Shubik power index for voter *i*. The SSPI of voter *i* is


$$\begin{array}{l} \phi_{i}=\underset{S\subseteq N\backslash \left\{i\right\}}{\sum }\frac{|S|!\left(n-|S|-1\right)!}{n!}\left[v\left(S\cup \left\{i\right\}\right)-v\left(S\right)\right] \end{array}$$ (78)

Modern contributions include algorithmic improvements, approximation schemes, and applications in multiagent decision-making (Aziz et al., 2007; Bachrach et al., 2010).

### 7.2 Aggregated ranking systems

Aggregated ranking systems combine multiple individual rankings into a single collective ranking. Such systems play a central role in elections, recommender systems, sports analytics, and machine learning (Conitzer, 2010; Kaniovski and Zaigraev, 2011). Given the set of alternatives *A* = {*a*_1_, …, *a*_*m*_} and the set of voters *N* = {1, …, *n*}, where each voter *i* reports a strict ranking ≻_*i*_ over *A*, an aggregation rule produces a collective ranking ≻^*^. Equation 79 formalizes this aggregation rule from individual rankings to a collective ranking.


$$\begin{array}{l} f\left({≻}_{1},\ldots ,{≻}_{n}\right)={≻}^{*} \end{array}$$ (79)

In weighted preference environments, each voter may also carry a weight *w*_*i*_≥0, reflecting formal influence or reliability.

Aggregated ranking rules can be categorized according to the informational primitive they emphasize: (i) *positional* or score-based methods that assign points to ranks (e.g., Borda), (ii) *pairwise-majority* methods that derive outcomes from weighted head-to-head comparisons and the induced majority tournament (e.g., Condorcet and Copeland), and (iii) *distance-based* rules or optimization approaches that define aggregation as an optimization problem over the space of linear orders (Brandt et al., 2016). Representative instances are presented below.

#### 7.2.1 Weighted Borda count

In the Borda count, if there are *m* alternatives, the Borda score assigned by voter *i* to candidate *c* is defined by its rank position. Equations 80–82 formalize the unweighted Borda score, the voter-weighted Borda score, and the total weighted Borda score of each candidate:


$$\begin{array}{l} {\text{Borda}}_{i}\left(c\right)=m-{\text{rank}}_{i}\left(c\right) \end{array}$$ (80)

With a voter weight *w*_*i*_, the weighted score becomes:


$$\begin{array}{l} {\text{WeightedBorda}}_{i}\left(c\right)=w_{i}\cdot \left(m-{\text{rank}}_{i}\left(c\right)\right) \end{array}$$ (81)

The total score of each candidate is:


$$\begin{array}{l} \text{Score}\left(c\right)=\overset{n}{\underset{i=1}{\sum }}w_{i}\cdot \left(m-{\text{rank}}_{i}\left(c\right)\right) \end{array}$$ (82)

The computational and strategic properties of Borda-based rules have been studied extensively, including manipulation, robustness and approximation guarantees (Xia and Conitzer, 2008; Caragiannis et al., 2017). Recent work emphasizes its suitability for large-scale and noisy environments, particularly in computational and machine learning contexts.

#### 7.2.2 Condorcet methods with weighted preferences

Condorcet methods evaluate alternatives through pairwise weighted comparisons. Equations 83–85 formalize the weighted pairwise victory score, the weighted majority margin, and the Condorcet-winner condition. Candidate *a* defeats *b* if:


$$\begin{array}{l} W_{a>b}=\underset{i:a{≻}_{i}b}{\sum }w_{i} \end{array}$$ (83)

For any *a, b* ∈ *A*, the weighted majority margin is defined as:


$$\begin{array}{l} M\left(a,b\right)=\underset{i:a{≻}_{i}b}{\sum }w_{i}-\underset{i:b{≻}_{i}a}{\sum }w_{i} \end{array}$$ (84)

This induces a weighted majority graph (tournament) *G* = (*A, M*), where edge orientation and magnitude encode pairwise dominance.

An alternative *a* is a *Condorcet winner* if


$$\begin{array}{l} M\left(a,b\right)>0\;\forall b\in A\backslash \left\{a\right\} \end{array}$$ (85)

Condorcet winners need not exist due to majority cycles, motivating rules that select winners/rankings from the tournament structure. Weighted majority graphs and their associated algorithmic questions (e.g., winner determination and manipulation) are central topics in the computational social choice literature (Brandt et al., 2016; Obraztsova et al., 2011).

#### 7.2.3 Copeland method

The Copeland rule assigns each alternative a score based on its pairwise victories and defeats. Equation 86 formalizes candidate A's Copeland score. Specifically, candidate A's Copeland score is defined as:


$$\begin{array}{l} C\left(A\right)=|\left\{B:A\text{ defeats }B\right\}|-|\left\{B:B\text{ defeats }A\right\}| \end{array}$$ (86)

Weighted Copeland variants naturally arise in environments with unequal voter influence using weighted majority comparisons. This method is closely related to tournament theory, revealing strong connections to majority cycles and dominance relations (Brandt et al., 2016).

#### 7.2.4 Kemeny-Young optimal aggregation

Distance-based ranking systems define aggregation as an optimization problem over the space of all linear orders. Equation 87 formalizes the Kemeny-Young optimal aggregation rule as the minimization of total weighted Kendall tau distance. The Kemeny–Young rule selects the ranking ≻^*^ minimizing the total weighted Kendall tau distances:


$$\begin{array}{l} {≻}^{*}=\arg\underset{≻}{\min}\overset{n}{\underset{i=1}{\sum }}w_{i}\cdot d_{\tau }\left({≻}_{i},≻\right) \end{array}$$ (87)

where *d*_τ_ measures the number of pairwise disagreements between rankings.

This formulation admits a strong normative interpretation, as it corresponds to maximum-likelihood estimation under a noisy ranking model. Despite its NP-hardness, recent research has developed fixed-parameter tractable algorithms, integer programming formulations, and heuristic approximations suitable for real-world datasets (Betzler et al., 2009; Conitzer et al., 2006; Ali and Meilă, 2012).

### 7.3 Importance of unequal voting weights

Unequal voting weights are prevalent in contemporary collective decision environments in which formal influence is allocated proportionally to stakeholder size, institutional responsibility, resource contribution, or estimated competence. Such asymmetries arise both by design—to reflect heterogeneous entitlements or obligations—and by necessity, when equal weighting would misrepresent the underlying distribution of authority or expertise. As a result, weighted voting constitutes an indispensable modeling abstraction for capturing how decisions are actually formed in a wide range of socio-technical and institutional settings.

Prominent examples include the voting architecture of the Council of the European Union, where qualified-majority rules combine member-state and population thresholds to balance representation across states and citizens (Le Breton et al., 2012). Analogous heterogeneity appears in multiagent decision frameworks, where agents may differ in reliability or delegated authority, and in machine-learning ensembles, where classifier reliability motivates weighted aggregation (Kuncheva, 2014). These applications show that unequal weighting is not a marginal variant of voting theory but a core requirement for faithful representation and robust aggregation.

These applications show that unequal weighting is not a marginal variant of voting theory but a core requirement for faithful representation and robust aggregation. Consequently, weighted models require rigorous mathematical and computational analysis to quantify effective influence, diagnose potential inequities, and assess robustness under strategic behavior and noise. In this sense, power indices and ranking aggregation rules are complementary instruments: the former evaluate how weight translates into decisiveness, while the latter specify how heterogeneous preference information is fused into collective choices.

### 7.4 Computational and theoretical considerations

Aggregated ranking systems exhibit a fundamental trade-off between expressive power, computational tractability, and resistance to strategic behavior. While score-based rules are computationally efficient, they may violate Condorcet consistency. Conversely, Condorcet-consistent and distance-based methods often incur significant computational complexity.

Recent advances in computational social choice focus on approximation algorithms, parameterized complexity, and learning-based aggregation mechanisms, particularly under weighted preference models (Brandt et al., 2016; Caragiannis et al., 2017). These developments highlight the growing relevance of aggregated ranking systems in modern decision-making frameworks where both preference intensity and voter heterogeneity must be accounted for.

## 8 Conclusions

This review has approached global university ranking as a methodological and algorithmic problem: how heterogeneous, multi-dimensional evidence (research production and impact, internationalization, teaching proxies, resources, societal engagement) is transformed into outputs that are both comparable and decision-relevant. A central conclusion is that there is no neutral, universally valid notion of “university quality” that can be losslessly compressed into a single scalar. Any ranking system operationalizes a particular evaluative model by selecting indicators, imposing directionality and normalization, adopting aggregation rules or dominance relations, and thereby encoding—explicitly or implicitly—a value-laden conception of excellence (Hazelkorn, 2015; Moed, 2017; Marginson, 2014). Consequently, differences between established global rankings (ARWU, THE, QS, U.S. News, CWUR) and alternative methodologies are not merely technical; they reflect distinct and often contested interpretations of what counts as institutional performance.

Even the most sophisticated ranking model cannot compensate for systematically biased or low-quality input data. For this reason, methodological implementation must be complemented by equally explicit commitments to data governance: clear indicator definitions, verifiable reporting protocols, transparent handling of missingness, and robustness checks that communicate when differences are not substantively reliable. Without such practices, ranking methods risk becoming precisely computed artifacts that project false precision onto noisy, incomplete, or strategically reported evidence.

Within this landscape, bibliometrics functions as a foundational evidentiary infrastructure for ranking because it offers scale, external observability, and a systematic basis for comparing research activity across large populations of institutions. At the same time, bibliometric indicators do not measure a single underlying construct directly: coverage differences across databases, field-specific citation norms, language and discipline biases, affiliation disambiguation, time windows, and counting conventions can all affect institutional estimates and shift the resulting ordering (Piro and Sivertsen, 2016). For this reason, bibliometrics should be treated primarily as an indicator system that requires normalization, documentation, and cautious interpretation.

The major global rankings reviewed in this article largely instantiate the weighted composite paradigm: raw indicators are transformed into comparable scores through normalization and, in some cases, non-linear compression, and then aggregated using fixed weights. The principal strength of this paradigm is communicative efficiency. It produces a single overall score and a complete rank table that is easy to disseminate and integrate into institutional narratives, policy discourse, and stakeholder decision-making. Moreover, certain transformations can improve robustness by dampening the impact of extreme values and stabilizing year-to-year volatility; ARWU's square-root compression is a prominent example whose core structure has been reconstructed and validated in the scientometric literature (Docampo, 2013). Nevertheless, the costs are substantive. Fixed weights and compensatory aggregation implicitly authorize trade-offs between dimensions that are rarely justified as universally valid, allowing excellence in one domain to offset weakness in another even when such substitution is conceptually questionable (Hazelkorn, 2015; Dill and Soo, 2005). In addition, indicator choices can systematically privilege older, well-resourced, research-intensive institutions and citation-dense fields, while undervaluing missions and disciplines whose outputs are less well-captured in standard bibliometric infrastructures (Piro and Sivertsen, 2016). Finally, partial opacity in data-cleaning routines, normalization constants, and proprietary adjustments limits full reproducibility in some systems despite increasingly detailed methodological disclosures (Times Higher Education, 2025; QS Quacquarelli Symonds, 2025).

Recent sustainability-oriented frameworks such as the QS Sustainability Rankings and UI GreenMetric reinforce this conclusion. By foregrounding environmental impact, social responsibility, and governance, they extend ranking logics into new domains while making the normative character of indicator selection even more explicit. Their rise suggests that future methodological debates will concern not only how indicators are normalized and aggregated, but also which institutional missions are considered worthy of measurement in the first place (Buckner and Zhang, 2025; Alberti et al., 2025).

Dominance-based methods (Pareto and Skyline families) provide a conceptually different response to the same need for cross-institutional comparison. Instead of imposing global trade-offs through weights, they adopt an order-theoretic semantics in which an institution is deemed superior to another only when it is at least as good on all criteria and strictly better on at least one. This paradigm is especially attractive in bibliometric evaluation because it preserves the multi-dimensional character of performance and avoids the normative compression of heterogeneous evidence into a single number. It naturally yields tiers or fronts of excellence, aligning with the view that many institutions are incomparable when they excel on different dimensions (Stoupas and Sidiropoulos, 2021; Stoupas et al., 2021). However, Skyline outputs often become large in high-dimensional spaces, which can reduce practical usability and motivate refinements such as skyline layering, dominance-based scoring, graph-based propagation, and ideal-point proximity measures (Chester and Assent, 2015; Martin-Nevot et al., 2025). These refinements improve discrimination, but they reintroduce modeling assumptions at a secondary stage and must therefore be justified with respect to what they measure and how they affect interpretive fairness.

Closely related to the objective of avoiding false precision are dashboard/profile systems and tier/threshold frameworks. Approaches such as U-Multirank, CHE-style groupings, and the CWTS Leiden Ranking shift the evaluative emphasis from producing a single global ordering to publishing multi-indicator profiles, indicator-level documentation, and categorical performance bands, often encouraging “like-with-like” comparison and discouraging over-reading of marginal differences (European Commission, 2014; European Union, 2015; DAAD, 2025; CWTS Leiden Ranking, 2025a). Their principal contribution is epistemic and communicative: they replace the claim of a universal best institution with dimension-specific information and structured comparability. Yet these systems do not eliminate normativity; the selection of indicators, normalization choices, grouping logic, and thresholds remain consequential design decisions. Moreover, the absence of a single scalar output can conflict with stakeholder demand for a simple rank table, which partly explains the persistent dominance of composite global rankings (Moed, 2017). In this context, uncertainty-aware reporting is particularly important: by incorporating stability intervals or inference-based classification rules, evaluation can avoid imposing spurious orderings when institutions are statistically indistinguishable (CWTS Leiden Ranking, 2025a; Abramo et al., 2018).

Frontier and efficiency-based methods (DEA and FDH) reframe ranking by treating universities as decision-making units and evaluating performance relative to an empirically constructed best-practice production frontier. Instead of aggregating indicators with fixed exogenous weights, these models assess how efficiently inputs (e.g., staff, expenditures) are transformed into outputs (e.g., graduates, publications, citations), yielding efficiency scores, peer benchmarks, and improvement targets. Their strength lies in managerial interpretability and in their explicit benchmarking logic, which naturally supports tier-like interpretation (frontier vs. non-frontier, or multi-frontier layering) (Johnes, 2015; Emrouznejad and Yang, 2018). However, they are not assumption-free. Results depend on orientation (input vs. output), returns-to-scale assumptions, variable selection, robustness to outliers and noise, and the dimensionality problem whereby too many indicators can inflate the number of efficient units (Agasisti and Johnes, 2015; Simar and Wilson, 2013). Accordingly, efficiency-based rankings should be interpreted as transparent, model-based benchmarks under stated assumptions rather than as direct substitutes for a universal rank table.

Latent-dimension approaches (PCA, factor analysis, multi-block reduction, and Bayesian latent-variable models) occupy an intermediate position. They typically preserve the production of a scalar score but estimate the effective aggregation structure from the data rather than imposing it a priori. Their contribution is twofold. First, they have diagnostic value: by revealing dominant latent dimensions in the indicator space, they can show how published composite rankings conflate distinct constructs, such as size-driven output and elite top-end performance (Dehon et al., 2010; Selten et al., 2020). Second, Bayesian and latent-trait formulations naturally support uncertainty-aware comparisons by producing posterior distributions over latent institutional quality and enabling probabilistic ranking statements (Claassen, 2015). Nonetheless, “weight-free” is best understood as rhetorical rather than literal: explicit weights are replaced by implicit statistical loadings that can be dominated by prevailing covariance structures reflecting size, disciplinary composition, or historical prestige rather than a policy-relevant conception of performance (Brankovic et al., 2018; Moed, 2017). In addition, estimated loadings are sample-dependent and sensitive to scaling choices, limiting cross-system and longitudinal comparability.

Computational social choice models, including weighted voting games and aggregated ranking rules, provide a rigorous formal vocabulary when the inputs are preference rankings or when multiple ranking sources must be fused into a collective order. Methods such as Borda, Condorcet/Copeland, and Kemeny-Young come with well-studied axiomatic properties and make explicit the trade-offs between tractability, robustness, and normative desiderata (Brandt et al., 2016; Conitzer, 2010; Betzler et al., 2009). Their relevance to university ranking arises when one treats different rankings, expert panels, or stakeholder groups as “voters” whose evaluations must be aggregated. Yet these methods also underscore that aggregation is intrinsically normative and often computationally complex, and that strategic or paradoxical outcomes can arise even when the inputs are already ordinal rankings.

Overall, this article argues that responsible bibliometric ranking is better served by methodological pluralism than by the search for a single universally superior formula. Composite rank tables maximize usability but embed strong normative assumptions and compensatory trade-offs (Hazelkorn, 2015). Dominance-based methods and profile/tier frameworks preserve multi-dimensional semantics and reduce false precision, but they provide outputs that are less aligned with the demand for a complete total order (Moed, 2017; Stoupas et al., 2021). Efficiency approaches capture distinct, interpretable dimensions—productive efficiency and relational influence—under explicit modeling choices (Johnes, 2015). Latent-dimension approaches provide parsimony and, in Bayesian variants, uncertainty-aware ranking, but remain sensitive to scaling, sampling, and interpretive validity (Brankovic et al., 2018; Claassen, 2015).

For university administrators, the central implication is that ranking systems should not be treated as interchangeable measures of overall institutional quality (Hazelkorn, 2015; Piro and Sivertsen, 2016). Composite rankings may be useful for external visibility, broad positioning, and communication, but they should be interpreted cautiously, especially when small score differences are overread as substantively meaningful (Saisana et al., 2011). By contrast, dashboard and profile-based systems are more appropriate for diagnostic use, peer comparison, and the identification of specific strengths and weaknesses across dimensions. Efficiency-oriented approaches such as DEA and FDH can support benchmarking, peer-target setting, and improvement planning, while latent-variable approaches can support strategic reflection, provided that their assumptions and limits are made explicit. In practice, ranking results are most useful when they inform institutional self-understanding and selective benchmarking, rather than being converted into a single strategic target (Hazelkorn, 2015; Rauhvargers, 2013).

Ranking methodology is never purely technical: because rankings function as governance instruments, choices about indicators, aggregation, and output structure also shape institutional incentives and patterns of adaptation. For that reason, alternative models should be evaluated not only in terms of formal elegance or statistical properties, but also in terms of the kinds of organizational behavior and policy response they are likely to induce (Espeland and Sauder, 2007).

Table 5 summarizes the main ranking families in terms of their institutional uses and interpretive cautions.

**Table 5:** Practical interpretation of ranking families for institutional use.

Ranking family/approach	Best use for university management	What should not be inferred	Typical strategic value
Composite rankings	External positioning, communication, broad visibility	Not a neutral or exhaustive measure of overall institutional quality	Reputation monitoring and external benchmarking
Dashboard/profile systems	Internal diagnosis, like-with-like comparison, multidimensional review	Not a basis for forcing universities into a single total order	Identification of strengths and weaknesses across dimensions
DEA/FDH	Efficiency benchmarking, peer target setting, input–output analysis	Not a direct measure of prestige, overall excellence, or universally valid performance	Resource-use evaluation and improvement targeting
Dominance/skyline methods	Multidimensional comparison without fixed trade-offs	Incomparability is not a defect, but a substantive result	Recognition of non-compensatory strengths and heterogeneous profiles
Latent-variable methods	Structural diagnosis, reduced-dimension synthesis, pattern detection	Results are sample-dependent and not always directly interpretable as institutional quality	Detection of underlying performance dimensions

In brief, the most coherent direction for bibliometrically informed university evaluation is not to replace one composite indicator with another, but to pair methods with questions and to accompany any scalar ranking with uncertainty reporting, sensitivity analysis, and complementary representations (fronts, layers, groups, and profiles) that preserve the multi-dimensional character of institutional performance. In this sense, bibliometrics should function not as a definitive verdict, but as a transparent and reproducible evidence base for evaluation that is explicit about its assumptions and modest about the strength of its claims (Moed and Halevi, 2015; CWTS Leiden Ranking, 2025a). For institutional users, this implies that ranking results should be aligned with evaluative purpose, interpreted alongside multidimensional profiles, peer groups, and uncertainty, and not converted into a blind optimization target centered on a single composite score.
